# Antibiotic stewardship challenges in Madagascar’s poultry sector: informality, knowledge gaps, and market constraints

**DOI:** 10.1371/journal.pone.0362299

**Published:** 2026-11-06

**Authors:** Sophie Molia, Ilo Tsimok’Haja Ramahatafandry, Michel Rakotoharinome, Christian Lienhardt, Muriel Figuié, Alexandre Hobeika, Daouda Kassié

**Affiliations:** 1 CIRAD, UMR ASTRE, F-34398, Montpellier, France; 2 ASTRE, Université de Montpellier, CIRAD, INRAE, Montpellier, France; 3 Department of Science and Veterinary Medicine, University of Antananarivo, Antananarivo, Madagascar; 4 World Health Organization, Antananarivo, Madagascar; 5 Veterinary Services, Ministry of Agriculture and Livestock, Antananarivo, Madagascar; 6 TransVIHMI, Université de Montpellier, INSERM, IRD, Montpellier, France; 7 Department of Infectious Disease Epidemiology and Dynamics, London School of Hygiene and Tropical Medicine, London, United Kingdom; 8 MoISA, Université de Montpellier, CIHEAM, CIRAD, INRAE, Institut Agro, IRD, Montpellier, France; 9 CIRAD, UMR MoISA, Maputo, Mozambique; 10 Universidade Eduardo Mondlane, Centro de Analise das Politicas (CAP), Maputo, Mozambique; 11 CIRAD, UMR MoISA, F-34398, Montpellier, France; 12 CIRAD, UMR ASTRE, Antananarivo, Madagascar; 13 Pasteur Institute of Madagascar, Antananarivo, Madagascar; USP FMVZ: Universidade de Sao Paulo Faculdade de Medicina Veterinaria e Zootecnia, BRAZIL

## Abstract

Antibiotic resistance (ABR) is a major global public health threat, largely driven by the overuse and misuse of antibiotics. Tackling its animal health component is particularly difficult in low- and middle-income countries, where data and resources are limited. This qualitative study aims to explore antibiotic use (ABU) and its underlying drivers in poultry farms in Analamanga, Madagascar’s region with the highest poultry density. In April 2023, we conducted semi-structured interviews with 79 poultry farmers (13 backyard and 66 semi-intensive), 10 animal health professionals, and 10 agrovet shop sellers. Thematic analysis was used to examine farm characteristics, poultry health management, veterinary medicine supply and use, and interactions with poultry production stakeholders, in order to identify antibiotic stewardship challenges in Madagascar's poultry sector. Backyard and semi-intensive farmers shared several features: informality (absence of farm registration, rare cooperative membership, limited training), irregular profitability, easy access to antibiotics, recurrent antibiotic misuse (for example use in poultry viral infection treatment, first-line fluoroquinolone treatments), and limited veterinary inspection by competent authorities. However, the two farmer groups differed, as semi-intensive farmers had slightly greater knowledge of ABU and ABR than backyard farmers; they primarily consulted chick and feed suppliers for health advice and medicine purchase; and they widely perceived antibiotics as essential to maintaining poultry health and productivity. Practical roadblocks to invest in preventive measures or adopt effective antibiotic stewardship (ABS) were their narrow profit margins, limited access to credit, and weak bargaining power towards poultry product buyers. Tailored approaches such as supporting professionalization of poultry sector, building capacity of chick and feed suppliers to provide training in appropriate ABU and management of flocks, flock vaccination, deworming schedules, treatment record keeping and ABS principles may enhance antibiotic usage in the Madagascar poultry sector. Future research should build on this exploratory study, which used a convenience sample of stakeholders, by examining ABU trends and drivers in Analamanga with representative sampling and rigorous quantitative validation across Madagascar’s poultry sector.

## 1 Introduction

Antimicrobial resistance (AMR) is the ability of microorganisms such as bacteria, viruses and fungi to become resistant to the drugs designed to kill or inhibit them [1]. It can develop naturally over time but is accelerated by the overuse and misuse of antimicrobial agents in humans, animals, and agriculture. AMR has become a major global health threat responsible for an estimated 1.14 million human deaths worldwide in 2019 [2]. International organizations have raised the alarm about this crisis for more than a decade, underscoring the need for coordinated global action to combat AMR and preserve the effectiveness of antimicrobials for future generations [3,4]. Addressing AMR emergence and dissemination requires a series of measures including infection prevention and control, rational use of antimicrobials, enhanced surveillance, and development of new antimicrobials and alternative therapies [5]. National Action Plans (NAPs) on AMR have been established in many countries to coordinate efforts across various sectors, including human health, animal health, agriculture, and the environment, through the One Health approach [6]. These plans often prioritize antibiotic resistance (ABR), which is the most widespread and urgent component of the AMR crisis [2].

The fight against ABR in low- and middle-income countries (LMICs) presents a unique set of challenges shaped by structural, economic, and institutional factors [7,8]. Surveillance systems are often weak or absent, limiting the availability of reliable data on antibiotic use (ABU) and ABR [9]. Regulatory frameworks are poorly enforced, allowing antibiotics to be sold over the counter without prescription, particularly in rural areas where access to trained medical professionals is limited [7]. High burdens of infectious diseases, combined with deficient access to water, sanitation and hygiene mean that human populations tend to rely heavily on antibiotic use [10]. Moreover, substandard or falsified antibiotics are more common in LMICs due to weak regulation of pharmaceutical imports [11].

In the livestock sector of LMICs, the availability of reliable data is even more limited than in the human health sector, despite growing evidence of increased veterinary drug consumption driven by rising consumer demand for animal products [8]. Antibiotics are sometimes routinely used as a cheap “insurance” against diseases or for growth promotion [12]. Extension services are limited and mainly provided by the agrovet suppliers, leading to widespread misuse [13–16]. A key challenge in improving ABU lies in simultaneously enhancing access to veterinary drugs—in terms of diversity, quality, and availability, while also strengthening regulations governing their use [17]. Overall, addressing ABR in LMICs requires context-sensitive strategies that account for these intertwined biomedical, socioeconomic and political realities.

Madagascar is one of the ten poorest countries in the world [18]. Agriculture is one of the country's main economic sectors, with 78% of the population living in rural areas. Around 60% of families raise livestock as a source of income and for self-consumption [19]. Poultry farming is the most widespread, accounting for 46.1 million animals compared with 9.1 million cattle and 3.5 million small ruminants [20]. Although some intensive commercial poultry farms exist, mainly around the capital city Antananarivo, the majority of poultry production in Madagascar is characterized by either backyard or semi-intensive systems [21]. The government of Madagascar is committed to combating AMR and designed its AMR National Action Plan (AMR – NAP) [22] which was adopted as a government program following Advisory Note No. 198/2019-PM/SGG/SC of April 30, 2019. The AMR-NAP is based on five strategic areas, one of which is the strategic priority of promoting the rational use of antimicrobials.

Data and research on ABU and ABR in the poultry sector in Madagascar has so far been very limited [23–26]. Campylobacter from chicken neck skins sampled at slaughter in Antananarivo in 2005–2006 showed resistance to ampicillin (35.8%), erythromycin (18.3%), ciprofloxacin (5.5%) and nalidixic acid (3.7%) [23]. In 2016, 70% of samples from industrial poultry farms around Antananarivo harbored extended-spectrum beta-lactamase-producing (ESBL) Enterobacteriaceae, with high co-resistance to tetracyclines, trimethoprim/sulfonamide and nalidixic acid [24]. Phylogenomic analyses of ESBL-Ec isolates later revealed multiple transmission events between humans, poultry and the environment [24], and high ESBL-Ec prevalence in healthy pregnant women, farm chickens and the environment, with strains and ESBL-carrying plasmids circulating across all three sectors [25]. Finally, a study on drug vendors and pig and poultry smallholder farmers in the municipality of Imerintsiatosika identified three patterns of opinion on ABU. The “confidence in antibiotics” group was associated with the use of antibiotics for disease prevention, poor knowledge of ABR and low trust in alternatives to antibiotics. The “belief in alternative methods” considered preventive ABU to be a major problem and believed that alternatives, such as vaccines, were useful preventive methods. The last group, “moderate view of antibiotic use”, had a hazier opinion [26].

A gap in the literature remains regarding the actual ABU practices of poultry farmers in Madagascar and how these practices are embedded within the broader socio-economic context. This information is crucial for designing effective measures to control ABR. To address this gap, our study aimed to investigate: (i) how Malagasy poultry farmers manage flock health, with a particular focus on the motivations and methods behind antibiotic use; and (ii) how underlying factors—such as socialization into poultry husbandry, economic pressures, and interactions with other animal health stakeholders—influence ABU across different farming systems.

## 2 Materials and methods

Following previous studies [26–28], we conducted a qualitative study based on semi-structured interviews to obtain in-depth, nuanced and contextualised understanding of practices [29]. The study protocol was submitted to and approved by the Veterinary Services Directorate of the Ministry of Agriculture and Livestock. It was also submitted to the Madagascar National Ethics Committee in Life Sciences and Health, which confirmed that formal ethical approval was not required for this type of study. Additional information regarding the ethical, cultural, and scientific considerations specific to inclusivity in global research is included in the Supporting Information (S1 Checklist).

Study sites were selected purposively, in collaboration with Madagascar's Department of Veterinary Services, whose expert knowledge of the national poultry sector guided the choice of the Analamanga region. This region was selected because it combines a high density and diversity of poultry farming systems, ranging from backyard operations to large commercial farms. The study was conducted in four municipalities within the Analamanga region (Ambohijanaka, Antanetibe, Itaosy, and Ivato) to reflect the diversity of poultry production systems in the region (Fig 1). Selection criteria included the predominant type of poultry farming practiced in each municipality. In Antanetibe, a rural municipality near Mahitsy, poultry farming is intensive, with some farms managing up to 40,000 birds and operating at a higher level of mechanization than those in the other municipalities. In the peri-urban municipalities of Itaosy and Ivato, farms are also intensive but generally smaller in scale. These farms primarily produce broiler chickens for the local market. In Ambohijanaka, a mix of semi-intensive and family-run farms is present, with semi-intensive operations producing mainly broilers—averaging around 200 chickens per farm.

**Fig 1:**
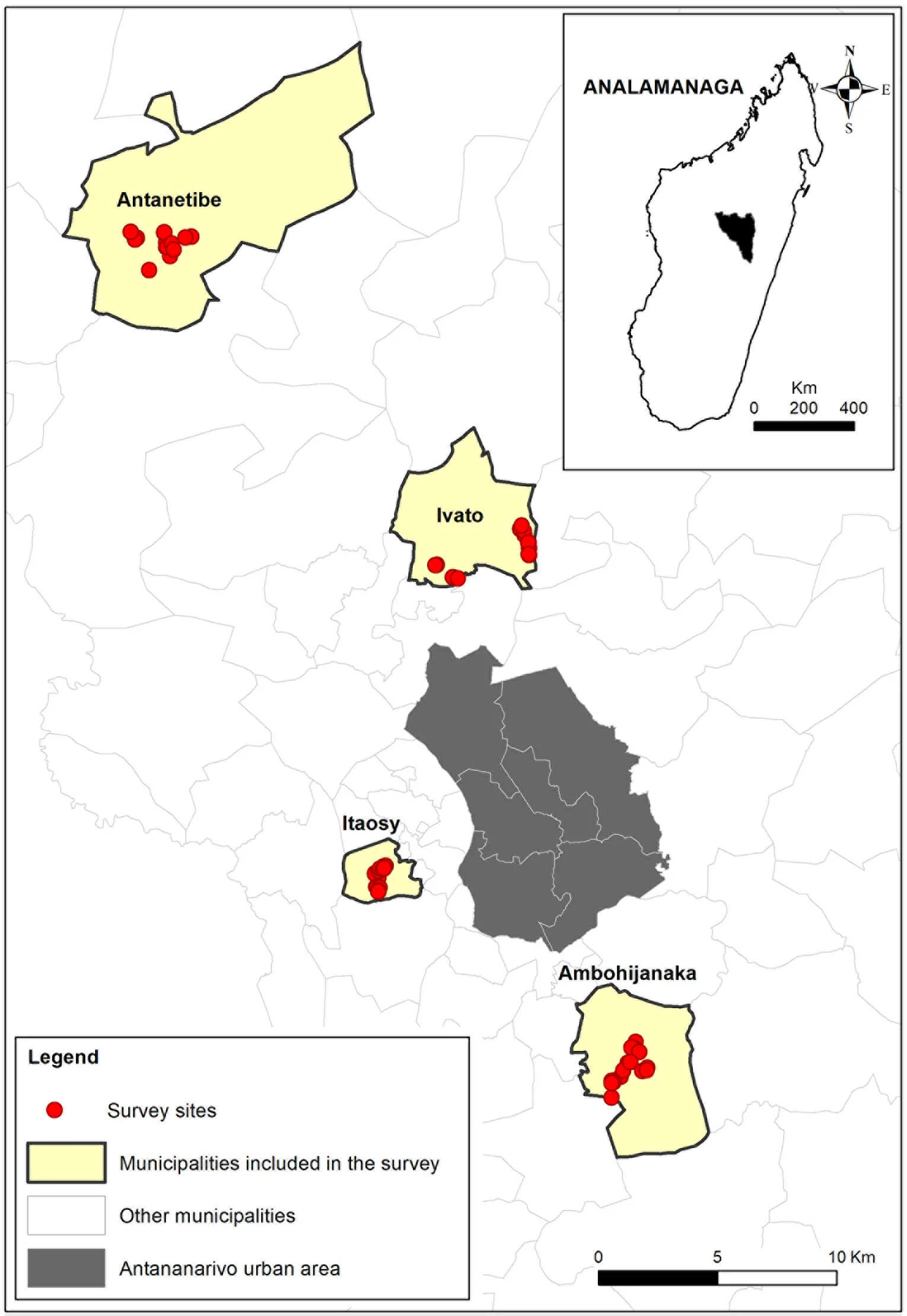
Location of survey sites (Source of administrative areas: https://data.humdata.org/dataset/cod-ab-mdg).

Farm categorization thresholds used in our study were based on previous studies [30–32]. “Backyard farms” were small-scale units (usually fewer than 50 birds), managed by household members with minimal infrastructure and mainly for self-consumption or a low input-low output sale logic. They are also known as family farms, traditional farms, small-scale farms or scavenging systems. “Semi-intensive farms” were intermediate systems, ranging from 50 to several thousand birds, with simple permanent structures, occasional hired labor, and production geared toward local markets. “Intensive farms” were large-scale, commercial operations (often over 10,000 birds) concentrated around Antananarivo, characterized by mechanization, salaried staff, and standardized veterinary protocols. Both semi-intensive and intensive farms have an entrepreneurial investment approach.

Within each municipality, the study targeted three main groups of respondents: poultry farmers, animal health professionals (including veterinarians, veterinary agents, and technicians), and staff from supply shops that sold veterinary drugs, herein designed as agrovet shops. We used the term “veterinary agents” for professionals who assist veterinarians in their daily tasks, particularly in clinics or practices. They may be involved in preparing for consultations, assisting with surgery, managing medication stocks, and providing information to animal owners. However, it should be noted that the term “veterinary agent” can be used more generally in Madagascar to refer to a veterinary paraprofessional or local animal health agent (APPSA or “agents de proximité en production et santé animals”). The aim of interviewing a diverse range of actors involved in the prescription, sale, and use of antibiotics in the poultry sector was to enable stakeholder triangulation, that is cross-validating ABU practices reported by farmers and exploring differences in attitudes, beliefs, experiences, and knowledge across actor groups.

Given the qualitative design of this study, sampling was not intended to be statistically representative but rather to capture a diversity of contexts, production systems, and stakeholder perspectives relevant to antibiotic use in poultry farming. Participants were selected using a combination of purposive and convenience sampling. Purposive sampling was used to ensure inclusion of animal health professionals and agrovet shops present in the municipalities and a balanced distribution of participants across the four municipalities. Convenience sampling was applied for recruitment of farmers, based on availability and willingness to participate. The Ministry of Agriculture and Livestock provided information on animal health professionals operating in the selected municipalities. In the absence of a formal sampling frame for agrovet shops and poultry farmers, we collaborated with local agricultural organizations, farmers’ associations, non-governmental organizations (particularly Agronomes et Vétérinaires Sans Frontières), local authorities (including fokontany chiefs, as fokontany represent the smallest administrative unit in Madagascar and serve as the primary governance structure at the local level), as well as veterinarians and veterinary agents to identify and engage relevant poultry farmers and agrovet shops for the study. Sample size was determined iteratively during fieldwork, with recruitment continuing until daily review of the transcripts showed no new themes emerged and data saturation was reached, that is when the ideas expressed in new interviews repeatedly mirrored those already identified in previous ones [33]. A total of 79 poultry farmers, 10 animal health professionals, and 10 individuals working in agrovet shops were interviewed.

Semi-structured interviews were conducted in Malagasy in April 2023 by a team of three trained interviewers, comprising two sociologists and one veterinarian. A local guide (a trusted intermediary identified by the chef de fokontany) accompanied the research team to facilitate communication and trust. In line with qualitative research methodology [34], semi-structured interviews were conducted using interview guides to facilitate open-ended discussions, allowing participants to freely express their experiences and perspectives on poultry farming and health management. Interview guides are provided in the Supporting Information (S2 Interview guides). All interviews were recorded using tablets equipped with the Kobo Toolbox platform. Each interview began with an introduction from the interviewer, including an explanation of the study’s objectives, the types of questions that would be asked and the respondent’s expected role—to freely share their views. This was followed by information on the estimated duration of the interview, the purpose of audio recording, the subsequent use of data, assurances of confidentiality and data security, and the respondent's rights: to withdraw from the study at any time without penalty, to stop the interview at any time, to skip any question, or to request that their responses not be used in the study. Once the participant agreed to proceed, signed informed consent was obtained.

Interviews with poultry farmers lasted an average of 40 minutes and followed a five-part interview guide. The first part collected general information about the participant through closed-ended questions on age, sex, education level, type of farming, and similar background data. The remaining four parts included open-ended questions covering: (i) a description of the farm’s main characteristics and the respondent’s background and role (e.g., farm owner or employee); (ii) animal health and the use of veterinary medicines; (iii) interactions with veterinarians, other advisors on poultry production, and animal health authorities; and (iv) the broader farm environment (e.g., chick and feed supply, the quality of which is known to affect poultry health and consequently veterinary drug use).

Interviews with animal health professionals lasted an average of 38 minutes and were guided by a four-part structure, consisting of open-ended questions on: (i) career path, current role, and work environment; (ii) antibiotic and medication use in the poultry farms they work with; (iii) norms, practices, and sanctions related to antibiotic use and resistance; and (iv) relationships with other stakeholders in the poultry production sector.

Interviews with agrovet shop staff averaged 39 minutes and also followed a four-part guide. These interviews explored: (i) background information about the respondent and the shop; (ii) sources of veterinary drug supplies and the proportion of antibiotics in total sales; (iii) knowledge, training, and awareness of norms and sanctions related to antibiotic use and resistance; and (iv) relationships with other actors involved in poultry production.

Throughout the fieldwork, observations were recorded and photographs taken to document and illustrate the different situations encountered.

Descriptive statistics, including minimum, maximum, averages, and frequencies, were calculated using Excel for both quantitative data and categorical variables. These were used solely to summarize respondent characteristics and contextualize the sample; no statistical inference was intended. For qualitative data, each interview was transcribed in Malagasy by the original interviewer, then translated into French. To ensure validity during translation, a cross-checking process was conducted whereby a bilingual researcher (third author) reviewed a subset of transcripts to verify accuracy and consistency between the original and translated versions. Thematic analysis was conducted following the approach outlined by Braun and Clarke [35]. Initially, a thorough reading of the entire dataset was carried out to identify relevant themes and sub-themes. A preliminary coding grid was developed by the first and last author and subsequently refined through exchanges with the other authors and through joint coding of a subset of 10 interviews. The final coding grids are provided in the Supporting Information (S3 Coding grids). Each interview was then coded according to the agreed upon coding grid using NVivo (version 1.7). Comparisons between farmer groups presented in the Results are descriptive and exploratory: they illustrate patterns and contrasts observed within our convenience sample and are not intended to support any claim of statistical significance.

## 3. Results

### 3.1 Description of respondents

Table 1 presents the sociodemographic characteristics of the 79 poultry farmers, 10 animal health professionals, and 10 agrovet shop workers interviewed, along with the municipalities where they were employed.

**Table 1:** Municipality and sociodemographic characteristics of the respondents.

Variables	Categories	Poultry farmer (n = 79)	Animal health professional (n = 10)	Agrovet shop (n = 10)
**Municipality**	Ambohijanaka	22	3	3
	Antanetibe	20	2	3
	Itaosy	18	2	1
	Ivato	19	3	3
**Sex**	Female	41	4	6
	Male	38	6	4
**Education level**	Primary	10	0	0
	Secondary	53	4	4
	Higher	16	6	6
**Age**	35 or under	38	4	6
	36-55	31	5	3
	Higher than 55	10	1	1

Among the 10 animal health professionals interviewed were 4 veterinarians, 1 livestock technician and 5 veterinary agents. The average experience of our respondent animal health professionals was 12.4 years (minimum 1.5, median 12, maximum 30) and they had all received training on veterinary drugs, whether during their study curriculum or during specific training organized by private veterinarians, agriculture projects or international organizations.

Among the 10 agrovet shop workers interviewed, 1 was a veterinary agent and 9 were salespersons. They had been in this position for an average of 6 years (minimum 0.6, median 4, maximum 19), and 7 out of 10 said they had received training on veterinary drugs, mainly from a private veterinarian but sometime from the Ministry or from the Maison du petit élevage (an inter-professional structure with association status).

### 3.2 Operational practices in poultry farms

#### 3.2.1 Variation in poultry farm types, flock sizes and production preferences.

In terms of farm type, 13 farmers managed backyard farms while 66 were involved in semi-intensive production systems. In the backyard livestock farming system, livestock production was managed by a family or a few family members, with most activities carried out by these individuals and minimal or no reliance on outside labour. These farms kept a small number of animals, allowing the family to handle all aspects of livestock management, including feeding, animal care, and infrastructure maintenance. Livestock farming was commonly integrated with other agricultural activities, such as crop cultivation or gardening, to make the most of available resources and reduce costs. Farmers in this system rarely reared broilers or laying hens; instead, they preferred Malagasy backyard chickens of the “Gasy” breed, in numbers ranging from 2 to 45 (with a median of 11 and an average of 13). Additionally, two farmers kept ducks or geese, and four farmers raised pigs, with herd sizes ranging from 2 to 8 animals. Poultry were housed either in a section of the family home (on the ground floor or upper level) or in a separate shed, generally constructed from earth, wood, and topped with a tin roof (Fig 2). Free-roaming of birds was common. None of the backyard poultry farmers belonged to a farmers’ association.

**Fig 2:**
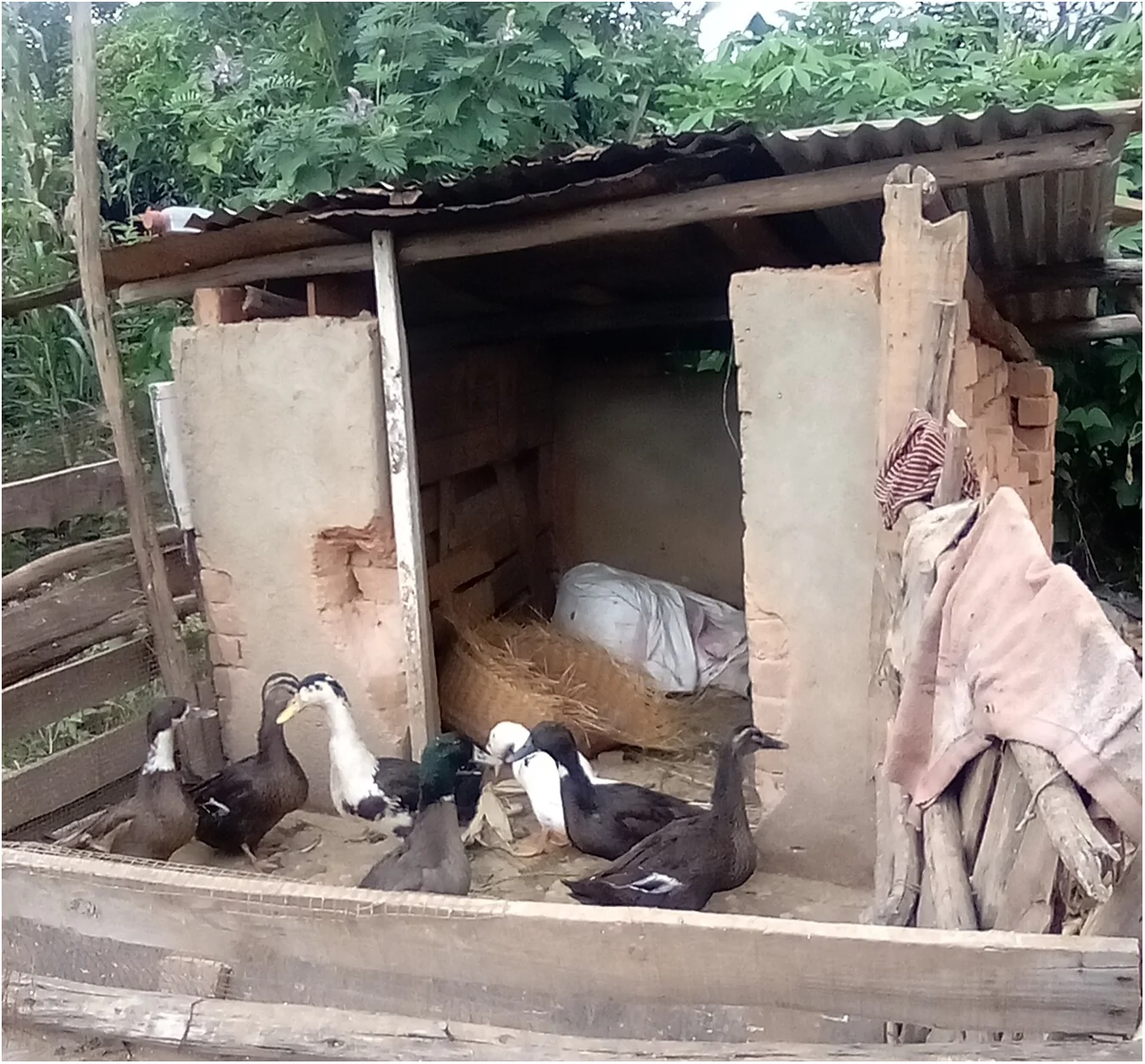
Backyard poultry shed.

Semi-intensive farming occupied a middle ground between backyard/family farming and intensive farming. It incorporated elements of both systems, aiming to enhance productivity while considering available resources and environmental constraints. Compared to backyard livestock farming, semi-intensive systems typically involved more developed infrastructure, such as permanent shelters, fencing, and watering systems. Building materials usually consisted of paving, cement or earth for the ground, bricks or cement for the walls, and tin or tiles for the roof (Fig 3). These farms were also more likely to employ staff, primarily responsible for feeding and watering the animals. Only 8 of the 66 semi-intensive farmers belonged to a farmers’ association. The types and minimum, median and maximum numbers of animals raised in semi-intensive poultry farms are presented in Table 2.

**Table 2:** Types and numbers of animals raised in semi-intensive poultry.

Species or breed	Number of farms raising this species or breed	Number of heads of each species or breed per farm		
		Minimum	Median	Maximum
Laying hens	27	15	400	6000
Broilers	44	20	100	3000
Gasy chickens	14	3	10	80
Other poultry	4	5	50	100
Pigs	22	1	4	400
Cattle	8	1	3	10

**Fig 3:**
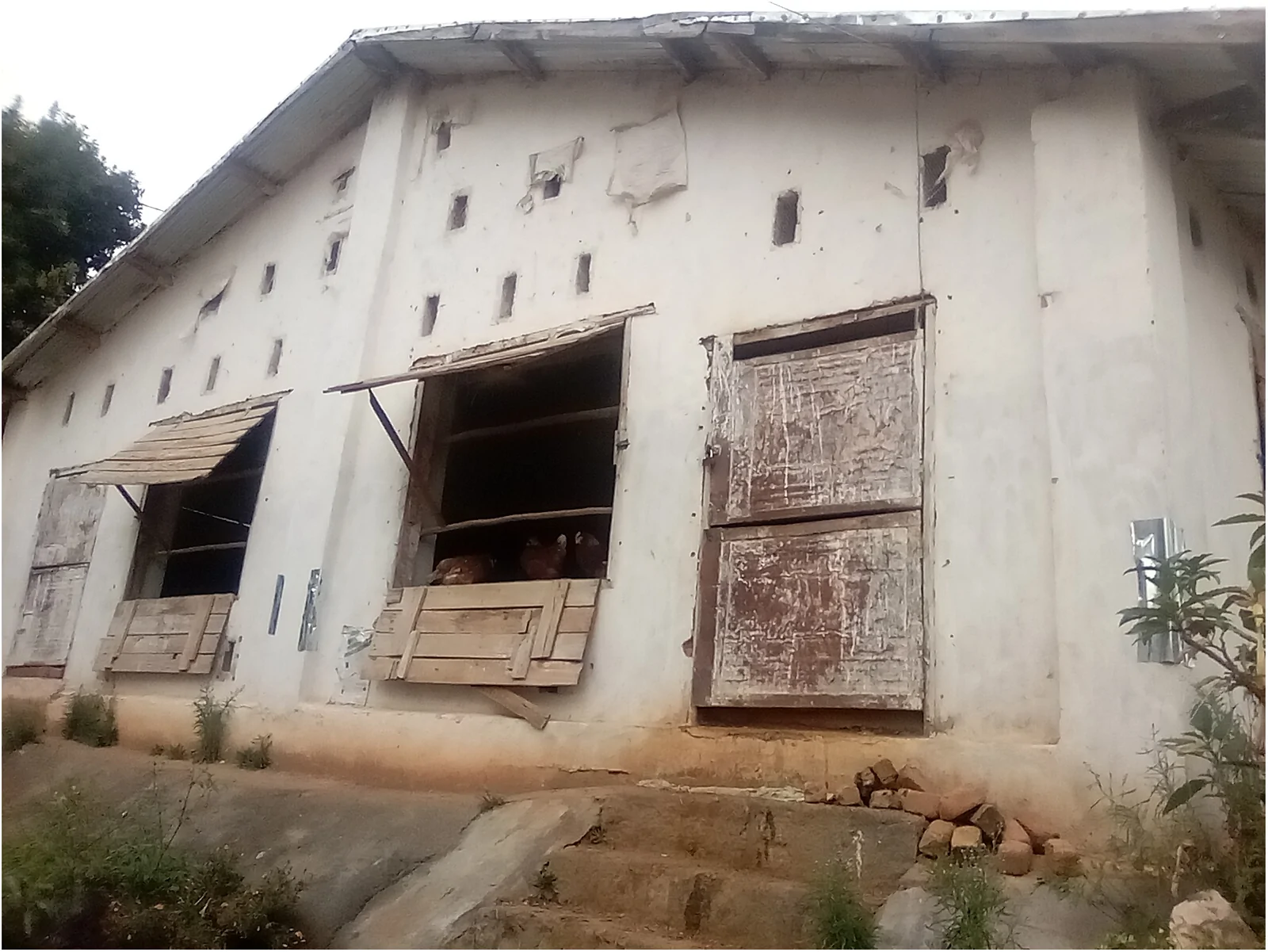
Building structure in a semi-intensive poultry farm.

Some poultry farmers preferred raising broilers due to the short production cycle, which enabled them to get a financial return in typically 45 days. Additionally, they considered broiler farming less complex and easier to manage compared to raising laying hens.

*“Compared to laying hens, broiler chickens are more quickly profitable.” (Farmer with 200 broilers)*

*“Production takes place over a short period of time, requiring only 35 to 40 days. Profits are obtained more quickly. I didn't consider raising laying hens because it takes a lot of time (5-6 months).” (Farmer with 110 broilers)*

*“Raising broiler chickens is a bit like receiving a monthly salary. I am already a broiler chicken seller, so it is easier for me to sell my products.” (Farmer with 150 broilers)*

*“So we started with laying hens and I sold the eggs myself, but the profit was too small and we couldn't do all the work on our own, so the laying hens didn't work out and we switched to broilers.” (Farmer with 270 broilers)*

*“There's a lot of risk with laying hens because if something goes wrong, we've already invested a lot of money and time, and it'll be hard to recover. Plus, they need a lot of care, it's too complicated.” (Farmer with 2600 broilers)*

Some poultry farmers preferred raising laying hens, either because it was a familiar trade within their family or community, or because they considered broiler farming to be less profitable. In one instance, a farmer chose to raise laying hens simply because their chick supplier only offered laying hen chicks.

*“We already tried with broiler chickens, but it wasn't very profitable. I'm already used to laying hens because I did it with my parents”. (Farmer with 600 laying hens)*

*“I copied what people around me were doing. Because they all raised laying hens” (Farmer with 600 laying hens)*

*“We have already raised broiler chickens before, but there are many people doing it, so the market is saturated.” (Farmer with 1500 laying hens)*

*“I used to raise broiler chickens, but the price of feed was very high while the “yield” was low, and the other person, who is a reseller, is the one who makes the profit.” (Farmer with 100 laying hens)*

Gasy chickens were raised in backyard farms and occasionally raised in some semi-intensive farms because they were highly valued and sold at higher prices than broilers, despite having a longer production cycle.

#### 3.2.2 Informality and multi-occupational nature of poultry farming.

For 4 backyard poultry farmers and 14 semi-intensive poultry farmers, poultry farming was their sole professional activity. For others, it was one of several occupations (typically two) including crop production, truck driving, shopkeeping, teaching, security work, or various forms of employment. One interviewee raised poultry not for income, but as a hobby. Education levels were lower among backyard farmers (primary: 3/13; secondary: 10/13) than among semi-intensive farmers (primary: 7/66; secondary: 43/66; higher education: 16/66).

Many poultry farmers began this activity after observing family members or, in some cases, members of their community raise poultry and earn an income from it. The initial funds to start poultry farming typically came from personal savings, income from other professional activities (either their own or their spouse’s), or loans from family members. Support from banks was rare, as interest rates were often considered prohibitively high. Only one farmer reported receiving government assistance, through the “Tosika Fameno” (“Bridging the Gap”) initiative—launched by the Malagasy government to support families following the Covid-19 lockdown.

Poultry operations typically began on a small scale, utilizing whatever space or land was available at the time. Expansion generally occurred only after sufficient profits were generated or when additional space was obtained. While poultry farming could sometimes yield enough income to finance major investments such as building a house, profits were often limited or irregular due to fluctuations in production performance (partly linked to disease) and market instability, particularly regarding input costs and the selling prices of chickens and eggs.

*“There was a time when we thought about making livestock farming our main job. But so far, we are not satisfied. It is true that it helps us send our children to school. One of our children is studying at university, and livestock farming helps us pay for tuition and registration fees. […] If the advertisements on the radio were true, livestock farming would provide for our needs. Until now, we have not considered it profitable, but as a resource in difficult times.” (Farmer with 200 broilers).*

*“Sometimes you get a lot and sometimes you get a little. The presence of middlemen is also a problem, and the price of chicken feed has become very expensive.” (Farmer with 200 broilers and 15 layer hens)*

Household members often played a crucial role in farming activities, in both backyard and semi-intensive systems. In most cases, the farmers themselves or their spouses were the primary decision-makers, overseeing farm management, feed sourcing and preparation, health treatments, and sales. When present, workers were typically responsible for routine tasks such as feeding and watering the poultry, operating lighting systems, removing manure, cleaning and disinfecting facilities and equipment, and collecting eggs. Farm managers often felt the need to closely supervise employees and were reluctant to entrust them with sensitive tasks, such as administering medications.

*“Things are starting to work now because I have competent staff. There was a time when I suffered heavy losses because I was often away and the staff did not pay attention to feeding the chickens.” (Farmer with 2600 broilers)*

Most farmers acquired their poultry farming skills informally, learning from relatives (such as parents, siblings, or uncles) already involved in the trade, by observing practices on other farms, or through personal experience. Only a minority of farmers (1 backyard farmer and 20 semi-intensive farmers) had received some form of training in poultry farming, offered by chick and feed suppliers or other training providers. Opinions on these sessions were mixed: some participants found them helpful, while others felt they were too brief or overly theoretical. Learning through books or online resources was rarely mentioned.

*“I didn't study and I didn't receive any training. The truth is that I still need more training because I don't feel up to the job. I've never asked anyone for advice before.” (Farmer with 48 broilers)*

*“I learned how to raise animals from my supplier. He was the one who advised me. But before that, one of my friends used to do it, and I always watched him and learned how to do it.” (Farmer with 60 broilers)*

*“I worked on the large farm La Hutte Canadienne for five years, and that's where I learned about animal husbandry. I only perfected my knowledge by taking training courses.” (Farmer with 1500 broilers)*

*“By following the training courses, I realised that I knew more than the trainers. [...] The training I received at Maison du Petit Elevage was very thorough, but there was one course I attended that was really not appropriate.” (Farmer with 2000 broilers)*

*“There are people who advertise on the radio saying that they give training courses, but they cost 35,000 ariary [7.5 USD], which I cannot afford.” (Farmer with 20 broilers)*

None of the farmers interviewed reported needing registration documents when they began poultry production. However, some mentioned either requesting authorization from the fokontany or being aware of tax obligations, although the vast majority of farmers said they were not registered and paid no taxes.

*“I haven't registered my farm yet because I don't want to deal with taxes.” (Farmer with 2600 broilers)*

When asked about their future aspirations, farmers mainly expressed a desire to increase the number of poultry, expand and improve housing facilities, diversify into other types of poultry, learn how to formulate their own feed, and use more professional equipment, such as replacing wooden feeders with more durable, purpose-built alternatives.

#### 3.2.3 Variability in input costs and quality.

Backyard poultry farmers typically sourced their Gasy chickens from other farmers, acquaintances or markets. In contrast, semi-intensive farmers obtained their chicks from a variety of large-company suppliers, with Agrival, Agrivet, Farmshop, Arbiochem, Avitech, LFL and Activo being the most commonly mentioned. The main chicken breeds cited included Manfonja, Avitec, Activo, Kinga, ISA Brown, and Hy-Line, although some farmers were unsure of the exact breeds they had purchased. Occasional shortages and quality issues (e.g., birth defects, poor growth, high mortality rate) were reported when sourcing chicks from suppliers. While some farmers frequently switched suppliers to secure better prices or to cope with supply disruptions, others preferred to remain loyal to a single supplier. Some suppliers provided data sheets detailing the vaccines administered to the chicks, though this practice was not consistent. In several cases, farmers were informed that the chicks had been vaccinated, but without clear information on which diseases the vaccines targeted. When specified, vaccinations were said to include protection against Gumboro, “plague” (a generic term commonly used for Newcastle disease), and/or infectious bronchitis.

*“A while ago, we bought some chicks nearby, but they were poor quality because after 45 days they only weighed one kilo, even though they ate a lot. We don't have a regular supplier of chicks.” (Farmer with 200 broilers)*

*“I always used Activo, but I might switch to Agrival to try them out. Because Agrival is offering a 10% discount. For example, if you buy 100, you get 10 extra. That way, if one chicken dies, you have enough to replace it.” (Farmer with 200 broilers and 10 Gasy chickens)*

In terms of feed supply, backyard poultry farmers tended to favor local shops or markets. Supply sources were more diversified among semi-intensive farmers, varying largely depending on price, quality and availability of feed. The cost of feed was very frequently cited as a major problem in poultry farming. Rising feed costs meant that it was more difficult to purchase feed (sometimes entailing decisions to reduce the flock size) and that profits diminished in a context where sale prices for eggs and broilers had remained the same or increased only slightly.

Feed brands such as LFL, Agrival and Feedmax enjoyed a good reputation, but were more expensive than homemade feeds. Feed purchased from suppliers could occasionally come with a technical data sheet, but such written documentation was rarely provided. Typically, broiler farmers gave starter feed for 15–17 days, then growth feed for 5–7 days, and finally finisher feed until the end of the production cycle. Due to fluctuating prices, quality problems or feed shortages, it was common for some farmers to switch feed suppliers regularly. In contrast, some farmers preferred remaining loyal to one supplier, either because they had a good experience with that supplier, or because this feed supplier was recommended by their chick supplier, or because they thought their loyalty would mean that they would still get supplied in case of shortages.

To increase their profit, farmers could decide to make their own feed for part or the whole production cycle rather than buying industrial feed from a supplier. These decisions depended on the quality and the prices of raw material, as well as the experience of the farmer in making feed.

*“LFL is very expensive and currently unavailable. There is a little bit every day or every other day, but nothing much. However, we don't have the money to buy ten or twenty kilos, just one or two kilos. Just the smell makes the chickens hungry, but it's expensive.” (Farmer with 200 broilers)*

*“It depends on the cost of the products; it's difficult to estimate the expenses, but at distributors it can be as much as 3,000 Ar [0.6 USD] per kg. We make it ourselves because it's more profitable for us. LFL is very good but very expensive, so we use LFL to start with, but we do the finishing ourselves.” (Farmer with 500 broilers)*

*“It is better to prepare the feed for the poultry yourself, because even if sales are not good, you will not lose money and you will still make a profit. But when it comes to feed, LFL is the best. When the corn is of poor quality, I use LFL.” (Farmer with 200 broilers and 10 Gasy chickens)*

*“I tried it first with 100 birds and tried all the different feed combinations, which allowed me to see what results I could expect from feeding the hens different diets. I was also able to find out the price ratios, which are based on price, time, and production weight. That's when I realized that it's more profitable to produce my own feed. I buy the ingredients from Arbiochem and make it myself.” (Farmer with 2600 broilers)*

#### 3.2.4 Limited bargaining power in market access.

Some backyard poultry farmers consumed their own chickens, while others did not, or only ate birds that were sick. They also sold chickens—either to fellow farmers in need of young birds, to community members, or to middlemen. These farmers generally expressed limited dissatisfaction with the prices they received. In contrast, semi-intensive farmers rarely reported consuming their own poultry, except for broken eggs or chickens that were undersized or had recently died. Many of them expressed frustration with the challenges of selling their products and the low prices they were often forced to accept. The market was seen as unstable, influenced by seasonal fluctuations in demand (higher during festive periods such as religious holidays), economic constraints affecting consumer spending, and competition from large-scale intensive farms releasing large volumes of chickens onto the market.

*“It is no longer very “profitable.” I think the reason for this is the problem and poverty of the Malagasy people, because eggs are not a staple food like rice, which you have to buy, but they are not necessary or essential.” (Farmer with 4500 laying hens)*

*“What I also notice is that broiler chickens are not a necessity for Madagascans, and consumers are very fickle. Personally, I don't introduce chicks in January and February because it's very difficult to sell broiler chickens at that time of year, and the same goes for September and October. It's best to increase the number of birds when festive events are approaching.” (Farmer with 2000 broilers)*

*“If you sell at a high price, you create a debt that will generally not be paid by customers. If you sell at a low price, you will earn money immediately, but neither the value nor the price you see will satisfy you. If you sell in the streets, you only sell two or three, it will take 10 days to sell all your chickens, and at the same time the food will be accounted for. If you sell to a wholesaler, he buys from you at a very low price. You can see that there is a problem with the chicken market. What we want is to have a fixed customer.” (Farmer with 200 broilers)*

*“The cost price for 1kg of meat is 8,000 ariary [1.7 USD], while at one point the market price for chicken meat was 6,500 [1.4 USD] per kilo. When we asked why the price had dropped, it turned out that a large farm had put 60,000 chickens on the market, so I didn't know how to sell my products.” (Farmer with 2000 broilers)*

Laying hen farmers either delivered their eggs to Antananarivo (or to other regions when demand in the capital was insufficient) or sold them to one or more middlemen. In contrast, broiler farmers reported a wider range of marketing channels. They sold chickens directly to local butcheries, markets, grocery stores, food stalls, and restaurants. They also sold directly to community members who visited the farm, to middlemen, retailers, or wholesalers from outside the community. In one case, a farmer used the chickens in a restaurant they personally owned. Broilers were typically sold once they reached a weight of 2.5 to 3.5 kg. The shorter the time required to reach this target weight (commonly around 45 days) the higher the profit margin.

*“There are already loyal customers, but there are also small buyers. I tried selling my products in supermarkets at one point, but the market was too corrupt; you have to know someone who works there to be able to introduce your products. My goal would be to sign a long-term contract with a buyer, but that remains difficult.” (Farmer with 2000 broilers)*

Some farmers also generated income by selling poultry litter to crop growers or gardeners. Others used the litter as fertilizer for their own crops. Finally, some simply discarded it nearby, allowing others to take it free of charge.

#### 3.2.5 Managing poultry health with varying advice source and dependence on veterinary drugs.

*Diverse sources of animal health advice*.

Poultry farmers generally had limited knowledge of diseases and were often unable to identify them by name. Instead, they described illnesses based on observable symptoms such as coughing, sore throat, white droppings, twisted neck (torticollis), the presence of worms, and a decline in egg production. Among the most commonly mentioned diseases were “plague” (a term commonly used to refer to Newcastle disease), “tety” (a Malagasy vernacular term usually referring to avian small pox), avian cholera, avian influenza and Gumboro.

When seeking advice on poultry health, backyard farmers typically turned to agrovet shop sellers, private veterinarians, and fellow farmers, particularly family members. In contrast, semi-intensive farmers primarily consulted technicians and veterinarians affiliated with chick and feed suppliers such as Arbiochem, LFL, Agrivet, Avitech, Activo, FarmShop, and Agrival, as well as private veterinarians when available locally. These animal health service providers were generally regarded by semi-intensive farmers as accessible, knowledgeable, professional, and capable of offering relevant guidance. Their services were seen as affordable, as they typically charged only for the medications they sold, with no or limited fees for consultations or travel. However, a minority of farmers found the cost of drugs to be excessive. Health management advice was often offered as an after-sales service bundled with chick and feed purchases, and in some cases, supplier technicians conducted routine visits even in the absence of reported health issues.

*“I call the Arbiochem technician when the farm needs monitoring, and it's free. Once I asked the technician to train my staff, and he did. […] I need the private vet mainly for the pigs. As for the poultry, I might need him one day because there are always unforeseen circumstances.” (Farmer with 2600 broilers)*

Some farmers did not use the services of animal health professionals for various reasons. In some cases, their poultry were never sick, or they had never felt the need to seek professional advice in the past. Others believed they had sufficient knowledge to manage common health issues on their own. Additionally, some farmers did not know any private veterinarians or only consulted them for other livestock species, particularly pigs.

*“In general, veterinarians don't really have much practical experience. […] If there is a case of disease, they are primarily interested in selling their medicines, especially if they have a practice and a medicine store at the same time. But in livestock farming, there aren't too many new things, so we've always been able to recognize what's going on. […] You observe the eggs to see if the size has decreased, if production has decreased, if several have died. And based on that, you analyze that something is wrong. If there are several deaths, we take a dead hen and a live hen to the state laboratory in Itaosy, where they perform an autopsy and we get the results of the analysis.” (Farmer with 500 laying hens)*

*“We never consult a [private] veterinarian, even though there are some in our municipality, because veterinarians are not familiar with the broiler chicken industry.” (Farmer with 500 broilers)*

In addition to veterinarians and technicians, some farmers also sought advice from fellow farmers or agrovet shops, although these sources were generally viewed as less reliable. As one farmer explained, “*We try what they recommend and keep only what works.”* Conversely, some farmers were reluctant to exchange advice with peers. Reasons included feeling sufficiently trained (such as in the case of one farmer who was also an agronomist), not feeling connected to other farmers, trusting only family or close friends, or a desire to protect their poultry farming “secrets.” Seeking information through online channels such as Facebook, Google, or other internet sources was rarely mentioned, as these were not commonly used for poultry-related guidance. Among the few online sources referenced, the Facebook page of the Arbiochem group was noted.

*Knowledge of prevention measures but implementation challenges.* Most farmers, especially in the semi-intensive sector, acknowledged the importance of preventive measures to maintain animal health. These measures included hygiene practices, vaccination and the provision of vitamins and dewormers. Hygiene practices included cleaning and disinfecting coops or barns, water troughs, and feeders; maintaining appropriate stocking density, ventilation, and temperature; using footbaths and/or changing footwear and clothing before entering the barn; restricting access to non-farm personnel; disinfecting and implementing all-out periods between production cycles; and isolating sick animals. The disinfection products most frequently used were Virunet, Despadac, and Cresyl.

*“We always disinfect every time a new batch arrives, using Virunet, which we repeat after 15 days. We treat them every week to prevent germs. If we hear that there are transmissible germs circulating, we use the disinfectant more frequently.” (Farmer with 110 broilers)*

*“We have installed a footbath at the entrance and disinfect it every week. When the construction work is complete, we will enclose the farm. Staff are required to remain in quarantine here during the cycle. The rest period is spread over 15 days: for the first 5 days, we clean and apply virucides; for the next 5 days, we use Despadac; and for the last 5 days, we use Cresyl, especially in the courtyard, and Virunet.”. (Farmer with 3000 broilers)*

However, these practices were not systematically applied across farms. Numerous hygiene lapses were either directly observed or detected in practices reported by farmers.

*“We are trying to follow the advice, but we don't know how to go about it. Their advice seems appropriate but not feasible, so it becomes inappropriate. For example, the chicken coop must be at least 2.5 meters high, and then you have to put up the ceiling, so how many bricks do you need to build that? And the area must be a certain size for a certain number of chickens, which is not feasible. We'll lose money if we follow this advice, because how many chicken coops will you build and how much will it cost, when how much do the chickens produce?” (Farmer with 400 laying hens)*

*“It's Avitech or LFL or Agrivet who send their technicians to build customer loyalty. But that doesn't suit me because they come back from other farms and then come to mine, and I don't like that.” (Farmer with 500 laying hens)*

In terms of vaccination, broiler farmers tended not to vaccinate because their chicks were assumably vaccinated before being delivered to the farm and because the production cycle was short. In contrast, the vast majority of laying hen farmers vaccinated their hens (or had them vaccinated) every one to two months and around three quarters of backyard farmers had their Gasy chickens vaccinated.

*“The vet said to vaccinate them for “tety” at 7 days old and for cholera and plague at 30 days, then give them a booster shot after 3 months if they are still there.” (Farmer with 45 Gasy chickens)*

*“We must follow the schedule carefully, respecting the dates for vaccinations, vitamins, and deworming.” (Farmer with 300 laying hens and 80 Gasy chickens)*

Vitamins were administered routinely, with AminTotal, Biovit, and Vitaflash as the most commonly mentioned registered names for vitamins. Combinations of antibiotics and vitamins were also frequent, with Covit, Tetracolivit, Colixivit, and Vitaseryl as the most commonly mentioned registered names for vitamin-antibiotic combos. These treatments were typically given when chicks arrived on the farm, during feed changes, when birds appeared weak or when egg production decreased.

*Widespread reliance on veterinary drugs.* When poultry fell ill, veterinary medicines were almost systematically used. Only a few farmers, mainly those practicing backyard farming, resorted to traditional remedies, such as chili, garlic, fruit tree leaves, or other plants. Some farmers attempted to adjust temperature and humidity, especially in cases of respiratory symptoms, before administering medication. Veterinary drugs were generally accessible either locally or in nearby towns, and could be bought from agrovet shops or veterinary drug deposits, markets, pharmacies, veterinary clinics, and feed and chick suppliers. Their cost was not usually seen as a barrier – only a limited number of farmers complained that medicines were too expensive – although farmers did not hesitate to travel to a different town to purchase the medicines for a cheaper price or to find a specific medicine they wanted. Most farmers did not keep a stock of veterinary medicines, although some stored poultry dewormers or treatments for other species like pigs. They preferred to purchase medicines as needed because stored drugs risked damage or expiration, and leftover medicines were typically discarded or burned. In contrast, a few semi-intensive farmers preferred to buy in advance the quantity of veterinary drugs required for an entire production cycle.

*“Yes, there was once a shortage of enrofloxacin, so we bought a large quantity. We also buy all the medicines needed for a batch each time they are introduced.” (Farmer with 3000 broilers)*

Farmers generally took care to keep veterinary drugs safely—keeping them out of children's reach, away from sunlight, and in dry areas. They primarily received advice on these medications from agrovet shop sellers, chick and feed suppliers, veterinarians, or specialist stores. No prescription was required to purchase veterinary drugs, nor was a farm-specific dosage prescription provided. Instead, farmers typically relied on the seller’s guidance and the information on the packaging, such as expiration dates, recommended dosages, and withdrawal periods. However, only a few farmers could recall the names of the drugs they used, with most remembering only a handful of vitamin and antibiotic registered names or molecules, such as AminTotal, Biovit, Vitaflash, Covit, Tetracolivit, Colixivit, Vitaseryl, enrofloxacin and colistin.

*Managing technical and medical issues with antibiotics amid limited ABR awareness.* When asked to define what antibiotics were, farmers replied in decreasing order of frequency that it was something that cured diseases, that prevented diseases or protected animals from disease, that strengthened animals, or they did not know or were not sure what it was. The main reason for using antibiotics was to treat sick animals and the most commonly treated illnesses included cough, “plague”, flu, colds, white stools, weakness, sore throat, torticollis, or drop in egg production. In the majority of cases, antibiotics were therefore given without any identified aetiology, simply based on a recommendation by either a veterinarian, a technician, an agrovet shop seller, or any acquaintance, in the absence of any examination of the birds. Some farmers treated their birds based on their experience, some gave antibiotics when they suspected an infection or observed illness symptoms, or when they were not sure what to do.

*“I don't need a prescription to buy medicine for my chickens. I just go to the seller and buy it. […] Yes, there is a veterinary medicine store in the commune. But we buy Covit from the feed seller.” (Farmer with 200 broilers and 10 Gasy chickens)*

*“When there is leftover water containing antibiotics, and when I clean their house, I give it to the ducks and chickens outside.” (Farmer with 50 broilers, 20 Gasy chickens and 5 other poultry)*

Other reasons for using antibiotics were not related to clinical signs and encompassed disease prevention, chick arrival at the farm, poultry growth promotion, change of feed, immunity improvement, or debeaking.

*“We give them Tetracolivit as soon as they arrive to relieve the stress caused by transport. […] This is described in the technical data sheet [provided by the chick supplier].” (Farmer with 150 broilers)*

*“Before vaccinating them against avian influenza and fowl pest, I give them this antibiotic. I vaccinate them every three months, so I inject them with the antibiotic every two months or two and a half months.” (Farmer with 10 Gasy chickens)*

*“On the 21st day, before they eat the “Finishing” feed, I give the chickens vitamins. The vitamin I use is Covit. Because that's what my older brother taught me.” (Farmer with 200 broilers and 10 gasy chickens)*

*“When they get avian flu, we give them tetracolivit. […] When we want to promote their growth, we give them this antibiotic. When we notice that they are a little behind in their growth, it gives them a good appetite.” (Farmer with 300 broilers)*

Some farmers believed it was not possible to produce chickens without antibiotics. Others had tried to reduce their use of antibiotics with more or less success. And others said they were not using antibiotics, including some who gave vitamins containing antibiotics and were not aware of it.

*"It's really important to give Tetracolivit for 5 days. We tried once not to give this treatment in order to produce more or less organic products, but the mortality rate was very high. And when we didn't use vitamin E, we noticed that many hens had difficulty standing up, so we always preferred to take preventive measures. If we don't use one of these treatments, we immediately notice the effects. So far, we can't do without antibiotics...". (Farmer with 70 broilers)*

*“It is only used if the chickens are sick, for example if they have a cold or cough, which are illnesses that occur in winter. […] When the feed is adequate and the ground on which they are kept is suitable, the chickens will always be healthy, and the number of chickens in a room should also comply with the standard.” (Farmer with 300 laying hens and 80 Gasy chickens)*

*“Since we started using sprays and footbaths, our use of antibiotics has decreased.” (Farmer with 4500 laying hens)*

*"We use antibiotics for treatment, but not for prevention. As far as we're concerned, we want to keep a certain reputation: the hens we produce are of good quality and don't give off too much odor." (Farmer with 2000 broilers)*

Regarding dosage, many farmers were able to specify the amounts they used—such as 1 teaspoon in 5–10 litres of water, or one packet in 10 litres—and typically administered the treatment for 3–5 days. However, some stopped giving antibiotics as soon as the birds showed signs of improvement.

*“When a chicken has a twisted neck, it must be treated with antibiotics. So the 5 days must be respected, but 3 days is the minimum if you happen to be short of money.” (Farmers with 200 broilers and 15 laying hens)*

*“I don't really read the instructions when I buy medicines, but I usually just look at the name and the date.” (Farmer with 12 Gasy chickens)*

When asked about withdrawal periods following antibiotic use, most semi-intensive broiler farmers showed an understanding of the concept and reported adhering to recommended intervals, ranging from 7 to 20 days. In contrast, laying hen farmers continued to sell eggs from hens undergoing antibiotic treatment. While some justified this practice by stating they used antibiotics without withdrawal periods for laying hens, others offered no explanation. A small number were unaware of what a withdrawal period entailed. Among backyard poultry farmers, knowledge of withdrawal periods was generally limited. They often linked non-compliance with the withdrawal periods to poor smell and taste of the meat. None of the farmers had ever been inspected by veterinary authorities concerning their use of antibiotics.

*“[Can the chicken be sold immediately?] According to the seller we asked, it doesn't matter. But in my opinion, it can't, because the medicine is still spreading in the chicken, it doesn't taste very good, like a cow after receiving an injection, its milk isn't good.” (Farmer with 20 broilers)*

About a quarter of farmers reported cases where antibiotic treatment was ineffective. When asked to explain this lack of efficacy, they cited possible reasons such as a mismatch between the antibiotic and the pathogen, underdosing, delayed administration, or poor bird immunity, while some admitted they did not know the cause. Notably, antibiotic resistance was never mentioned as a potential explanation. In fact, only around one in ten farmers had heard of antibiotic resistance, and among them, half either found the concept unclear or believed it only applied to humans.

### 3.3 Knowledge and views of animal health professionals

#### 3.3.1 Frustration with farmers’ antibiotic misuse and non-compliance with advice.

Animal health professionals often expressed a critical view of how poultry farmers managed flock health. They characterized small-scale farmers as doing whatever they wanted in terms of treatment (including in terms of antibiotics) and often going for what was cheapest. In contrast, larger farmers were described as more likely to follow supplier-provided technical instructions and to consult these suppliers when problems arose. Numerous instances of farmers misusing antibiotics were cited, including use without a proper disease diagnosis, use for growth promotion in broilers, preventive use, administration during feed changes, and overdosing.

*“There are people who use medications directly without knowing what they are. It is a medication, it treats, so they inject it without knowing the dose.” (Veterinary assistant with 18 years of experience).*

Animal health professionals also expressed frustration that farmers often disregarded their advice or first attempted to treat their poultry on their own. They reported that farmers typically sought professional help only after these initial efforts failed and the animals’ conditions had worsened. This delayed intervention made effective treatment more challenging, especially when farmers could not tell which first-line treatment they had used; and when recovery was not achieved, the farmers frequently placed the blame on the professionals.

*“There are times when it is very difficult to convince farmers and ranchers because they tend to believe rumors, but when they don't know what else to do, they come back to you.” (Veterinarian with 2.5 years of experience)*

*“It is common for people in Mahitsy to ask for a specific medication, and if it is not the same one, they will not buy it, especially if they are not the owner of the chicken but an employee. Even if it is explained that it is the “equivalent” of the medicine they are looking for, if it is not exactly the same as what they are looking for, they will leave. Sometimes, when we ask questions about the chicken's illness, we say that it is the right medicine for that illness, but they only ask for the medicine they want. In rural areas, this is common practice, not only with antibiotics but also with other medicines.” (Veterinary agent with 15 years of experience)*

When asked how antibiotic use among poultry farmers could be improved, animal health professionals emphasized the need for targeted training provided by the Ministry of Agriculture and Livestock. They recommended that farmers prioritize preventive measures such as vaccination, proper hygiene, temperature regulation, ventilation, and adequate nutrition. Professionals also advised against the use of antibiotics as a preventive measure or for treating mild symptoms, suggesting the use of vitamins instead in such cases. They stressed the importance of avoiding self-medication and urged farmers to consult veterinarians or trained technicians when problems arise, and to follow their guidance closely.

*“[How can we improve the use of antibiotics for poultry health?] Farm management is always important, including feeding, temperature, and everything else, such as deworming and vaccinations. When these things are under control, the use of antibiotics becomes rare, but they are still needed at certain times. However, they are used less frequently.” (Veterinary agent with 15 years of experience)*

#### 3.3.2 Gaps in knowledge of antibiotic use and resistance among professionals.

The animal health professionals demonstrated limited and incomplete knowledge of antibiotic use and antibiotic resistance. While most had heard of regulations governing antibiotic use, they were unable to provide specific details, likely due to the absence of enforcement as none had witnessed or heard of any sanctions related to the improper sourcing, use, or sale of antibiotics. Many confused regulatory norms with the instructions provided on medication leaflets. Some good practices were reported, such as prescribing or selling antibiotics primarily when poultry were sick, tailoring treatments to the specific disease and species, and, in certain cases, sending samples to a laboratory for diagnosis before prescribing antibiotics. However, several problematic practices and beliefs were also noted. These included the routine sale of critically important antibiotics as a first-line treatment due to their lower cost; the systematic use of antibiotics during viral infections to prevent secondary bacterial infections; and the belief that broilers could not be raised without antibiotics, leading to their routine administration. Conversely, Gasy chickens were considered more resilient and less in need of such interventions.

*“Giving medication depends on the farm, but it also depends on the disease. For example, if the disease is not serious, then a vitamin-enriched antibiotic is given to the chicken, as it contains antibiotics but is less strong.” (Veterinary agent with 15 years of experience)*

Some respondents were aware that antibiotic resistance could affect humans, but the consequences they mentioned were often limited to concerns about allergies and the need to thoroughly cook meat to avoid antibiotic residues. Notably, none of the professionals interviewed cited any examples of environmental impacts associated with antibiotic resistance.

*“If a medication is administered to an animal but it dies, the person should not eat it because the medication continues to spread throughout the animal. However, if the animal is thoroughly cooked, the potency of the medication can be inhibited. It can only be consumed if the animal is thoroughly cooked.” (Veterinarian with 23 years of experience)*

*“To prevent antibiotic resistance, my view is to frequently change the medications given to the animal.” (Veterinary agent with 15 years of experience)*

Overall, all the animal health professionals interviewed expressed a strong interest in receiving further training on antibiotic use and antibiotic resistance.

#### 3.3.3 Concerns about “non-professional” poultry health stakeholders.

Animal health professionals generally held negative views toward “non-professional” poultry health stakeholders, those without veterinary or technician qualifications and who were not supervised by trained professionals. These individuals were often seen as lacking adequate knowledge and, in some cases, as posing risks to both animal and human health. One respondent acknowledged their usefulness in rural areas, citing the shortage of trained professionals. Overall, relationships between professional and “non-professional” stakeholders were minimal or nonexistent. The regulation of “non-professional” actors, including unlicensed agrovet shop sellers, was viewed as the government’s responsibility, but respondents noted that such oversight was not enforced in practice.

*“How often do we complain to the government that there are too many people vaccinating without permission, too many people buying medicines illegally from suppliers?” (Veterinarian with 30 years of experience)*

*“It could upset the harmony within the community if I were to intervene. Besides, I am not an inspector for this locality; it is the State that must take care of matters such as this. Even with regard to warehouses, it is the Veterinary Services that must carry out inspections.” (Veterinary agent with 15 years of experience)*

*“If we prosecute these people, it will make the problem worse because, although they do not comply with the standards due to their lack of knowledge, they are also able to respond to people's concerns. These are things that I really see.” (Veterinary agent with 15 years of experience)*

### 3.4 Practices and perspectives of agrovet shop sellers

#### 3.4.1 Antibiotics sourcing and dispensing practices.

Agrovet shop sellers reported sourcing antibiotics from suppliers such as Arbiochem, MSP, Agrichem, and Laprovet, as well as from veterinarians, some of whom also owned or supervised the agrovet shops. Supply shortages or delivery delays were common, sometimes prompting sellers to change suppliers. The quality of medicines available on the Malagasy market was sometimes perceived as inconsistent. To ensure client satisfaction and encourage repeat business, sellers aimed to stock higher-quality antibiotics. They sought to achieve this by selecting trusted suppliers, prioritizing certain countries of production, and opting for medicines that were relatively more expensive, assuming these would be of better quality.

*“We can say that it is of good quality. We try it out and if it is bad, we don't buy it anymore. What I mean is that we use it first and if we are not satisfied, we stop buying from that supplier. […] I also look at the country of origin of the medicine; we only buy those made in France, not those from China.” (Seller with 8 years of experience)*

*“Veterinarians advise us on where to buy medicines. […] Buyers tend to go for the cheapest medicine. So when we buy medicines, we focus mainly on quality. We have to choose products that are inexpensive but of good quality.” (Seller with 1 year of experience)*

Overall, agrovet shop sellers had limited knowledge of the total quantity of antibiotics sold in their shops each month. When estimates were provided, they ranged from 60 to 300 bags of 100g antibiotics per month. Similarly, sellers found it difficult to quantify what proportion of the shop’s overall turnover was generated from antibiotic sales. The most commonly sold veterinary products included vitamins, vitamin–antibiotic combinations, and dewormers. Antibiotics were dispensed without requiring a prescription. When asked why, sellers explained that prescriptions were not considered necessary for animals, or that farmers knew which treatments their animals needed.

According to agrovet shop sellers, their customers included a mix of backyard and semi-intensive farmers (with the latter more likely to purchase higher-priced medicines), as well as technicians and veterinarians conducting field visits, and fighting cock owners. Some farmers arrived knowing exactly which medicine they wanted, while others sought advice. Sellers stated they were generally reluctant to dispense antibiotics without first gathering information about the type of bird, its living conditions, and observed symptoms. They noted that farmers often requested treatments that had worked for them or their neighbours in the past, without considering whether the current situation or disease was comparable. Such mimicking behaviour was described as common. In cases of uncertainty, sellers said they referred farmers to a veterinarian or directly contacted a supervising veterinarian. They also took into account the buyer’s purchasing power when making recommendations, sometimes offering smaller or lower-cost packages to farmers with limited financial means.

*“If they find good results after using a product, they demand the same product and will not accept an equivalent. For example, a customer demands the product name Covit even if they are offered its equivalent, Oxyvit forte.” (Seller with 10 years of experience)*

*“We have a veterinarian who supervises us, and apart from common illnesses, we refer customers to the veterinarian.” (Seller with 4 years of experience)*

#### 3.4.2 Poor understanding of antibiotic use and resistance.

The knowledge of antibiotic use and antibiotic resistance was poorer in agrovet shop sellers than in animal health professionals. About one third of them had received no training at all on veterinary drugs and learnt on the spot. They did not know about or thought there was no regulation governing antibiotic use, and they had never witnessed or heard of any sanction related to the improper sourcing, use, or sale of antibiotics. However, some of them knew that agrovet shops had to be inspected.

*“There are rules governing this, and state representatives come to inspect after a year's activity, and may be representatives of the fokotany or the commune. Then we start the necessary paperwork. We're currently preparing the documents for our deposit, because we've been here for almost a year.” (Seller with 1 year of experience)*

Most agrovet sellers were aware that preventive measures such as vaccination, proper hygiene, good nutrition, and the use of vitamins could improve poultry health and reduce the need for antibiotics. They also demonstrated some knowledge of appropriate dosages and withdrawal periods for antibiotic treatments. However, they reported dispensing critically important antibiotics as first-line treatments or experimenting with different antibiotics until one proved effective. Only four respondents had heard of antibiotic resistance, and their understanding was generally inaccurate, for example describing it as the animal’s body capacity to tolerate antibiotics.

*“First we try the antibiotics we have and change if they don't work. [...] You must not wait long, because some people wait until their animal is in danger for 3 or 4 days before coming and it's the strong antibiotic we give him.” (Seller with 19 years of experience)*

*“The most popular are Colivit and Enrofloxacin because they are the cheapest”. (Seller with 11 years of experience)*

*“The use of antibiotics depends on the case. For example, if a bird has angina, I use enrofloxacin.” (Seller with 10 years of experience)*

#### 3.4.3 Competition in the agrovet sector.

Agrovet shop sellers expressed concern over the rapid proliferation of agrovet shops (describing them as “popping up like hotcakes”) and the resulting increase in market competition. There was little to no interaction or collaboration with competitors, and many sellers voiced a desire for regulatory authorities to enforce a minimum distance between stores when granting new licenses, in order to limit market saturation.

## 4 Discussion

This is the first study to provide an in-depth account of the everyday practices of poultry farmers in Madagascar, both backyard and semi-intensive, and to situate these practices within their broader economic, social, and institutional contexts. The qualitative methodology was chosen for its proven effectiveness in capturing details and nuances, as demonstrated in previous studies [27,28,31,36].

### 4.1 Variations in ABU and its drivers across poultry sectors

Several ABU practices that deviate from established technical recommendations were observed in both backyard and semi-intensive poultry sectors. These included using antibiotics to treat viral infections, to boost birds’ immunity, or when uncertain about the appropriate course of action. In both sectors, treatments were sometimes under- or over-dosed, or prematurely discontinued once animals showed signs of improvement. Critically important antibiotics such as enrofloxacin and colistin were used as first-line treatments. These patterns align with findings from other Sub-Saharan African countries [31,37,39].

However, our findings also reveal differences in ABU and its underlying drivers between the backyard and semi-intensive poultry sectors. In the latter, we noticed farmers widely perceiving antibiotics as essential for maintaining poultry health and productivity, and antibiotics being frequently used for disease prevention (particularly upon chick arrival, during feed changes, vaccination, or debeaking) as well as for growth promotion in broiler production and enhancing egg yield in layer farms. Such practices and perceptions are commonly reported in commercial poultry systems across LMICs [39–44] and the link between the intensification of livestock farming and increased dependency to antibiotics has also been documented previously [45]. Semi-intensive farmers tended to rely more systematically on veterinary drugs than on alternative preventive strategies, such as infrastructure improvements (e.g., regular cleaning, disinfection, optimal stocking density, ventilation, heating), vaccination, and high-quality feed, all of which are recognized as effective in reducing ABU [46]. This reliance is likely driven by the relative affordability and ease of access to antibiotics, a pattern also noted in other LMICs [36,38,41,47]. In addition, limited financial resources, poor access to credit, and constrained market opportunities for poultry products restricted farmers’ ability to invest in preventive measures, thereby increasing disease risk. Economic vulnerability, particularly to fluctuations in input costs (notably feed) and market prices for poultry meat and eggs, led to precarious farm profitability, which has been described as reinforcing dependence on antibiotics [27,39,41,48,49].

Conversely, backyard poultry farmers used veterinary drugs less systematically than their semi-intensive counterparts. This difference is likely linked to the characteristics of backyard systems, which often involve indigenous chicken breeds perceived as more disease-resistant, minimal investment in housing, feed, and healthcare, and relatively low profit expectations [50,51]. Similar patterns were observed in North-central Nigeria, where 92.1% of free-range local flocks reportedly used antimicrobials rarely [39]. Moreover, the use of herbal remedies was more prevalent among backyard farmers than among semi-intensive ones, as documented in studies from Ghana [52] and Nigeria [53]. When backyard poultry farmers used antibiotics, certain aspects of their practices were generally weaker than those observed in the semi-intensive sector. They were more likely to seek advice from agrovet shop sellers rather than from more qualified professionals such as veterinarians or animal health and production technicians. Antibiotic choices were often based on price (typically opting for the cheapest option available) or on what had worked in other farms, regardless of whether the treatment was appropriate for their specific context. Additionally, backyard poultry farmers demonstrated lower awareness of key concepts such as withdrawal periods and the risks associated with antimicrobial residues and resistance, in line with results obtained in Nigeria [53] and Kenya [42].

### 4.2 The need for tailored ABR control measures

The excessive and inappropriate use of antibiotics in food animals is a key driver of ABR [54]. The findings of our study can inform targeted recommendations, particularly adapted to poultry farm types, to reduce the risk of resistant bacteria emerging and spreading within the poultry sector in the Analamanga region, the region with the highest density of poultry production in Madagascar. However, different or additional measures may be necessary for other regions of Madagascar, where local contexts vary. For instance, limited access to qualified animal production and health professionals was not an issue in our study region, but it is a common problem in many remote rural areas across Africa, which can hinder responsible antibiotic use [55–57]. The following recommendations are derived from our thematic analysis of the study findings, rather than directly reflecting suggestions made by respondents during interviews.

One of the primary challenges to address is the widespread lack of knowledge about animal health, ABU and ABR. It is a major factor for the poor ABU practices commonly observed in the Analamanga poultry sector. A first, necessary step is to communicate why some uses of antibiotics are problematic and which practical alternatives can be adopted. This information must be communicated to all stakeholders across the poultry value chain. Farmers, in particular, demonstrated the lowest levels of knowledge among our interview groups. Most were unable to identify diseases by name, relying instead on symptoms, had difficulty recalling the names or active ingredients of veterinary drugs, and could not define key concepts such as antibiotics, ABR, or withdrawal periods. Among our respondents whose profession involved selling antibiotics to poultry farmers, knowledge gaps were also evident among veterinarians and technicians and to an even greater extent, among agrovet shop sellers. These findings highlight the influence of education levels on how individuals perceive the risks associated with ABU and ABR [43].

Veterinarians and technicians employed by the major feed and chick supplying companies could be a key focus of ABU and ABR capacity-building and awareness efforts. There are several reasons for this. First, they are often the preferred advisors for semi-intensive poultry farmers, the highest users of antibiotics among our interview groups and those most inclined toward preventive ABU. Additionally, these professionals have more frequent and direct contact with semi-intensive farmers than private veterinarians, particularly with farm managers who are responsible for administering treatments. Finally, compared to private veterinarians, their business model is less dependent on antibiotic sales, as their income is diversified across a broader range of products such as feed, day-old chicks, and other poultry inputs. This makes them more likely to support initiatives aimed at reducing ABU, as the financial impact on their operations would be relatively limited.

The very liberal nature of the drug market with an absence of formal written prescriptions, is a common barrier to improving ABU practices [14]. Beyond raising awareness, feed and chick suppliers could be encouraged to provide clients with printed informational materials that include: i) an overview of common poultry diseases, including their aetiology (bacterial, viral, parasitic, etc.) and clinical signs; ii) a list of diseases covered by vaccinations administered to day-old chicks at delivery; iii) a vaccination and deworming schedule; iv) a logbook for recording production data, treatments, and feedback on treatment efficacy; v) practical husbandry recommendations, such as changing footwear before entering poultry houses, routine disinfection with virucidal agents, maintaining downtimes between flocks, installing footbaths, and fencing farm premises.

Private veterinarians, as well as sellers from agrovet shops, could be the second priority group for ABU/ABR capacity-building and awareness initiatives. Their training could align with that provided to veterinarians and technicians working for feed and chick suppliers but also include key messages tailored for backyard poultry farmers, a group they interact with more frequently. For instance, they could emphasize hygiene and litter management practices, given that backyard poultry farmers often share living spaces with their poultry, increasing the risk of microbial transmission and the spread of resistant genes [58].

A second key challenge is the lack of professionalisation within the poultry sector. Most farmers surveyed reported having no specific training and were not formally registered, with few engaging in collective activities or organized cooperatives. These gaps in formal structure are common across Sub-Saharan Africa [59,60] and significantly restrict opportunities for capacity building, quality monitoring, and negotiating bulk discounts on essential inputs such as feed, vaccines, and medicines. Additionally, the sector’s narrow profit margins, coupled with farmers’ weak bargaining power relative to poultry product buyers, further undermine their ability to engage in effective antibiotic stewardship [31,49]. Malagasy authorities and agricultural agencies could establish streamlined farmer registration processes to enable formal recognition, facilitate access to governmental and NGO support, and improve eligibility for bank credit. Farmers could also be encouraged to form or join cooperatives and producer associations. These organizations can play a crucial role in delivering tailored capacity-building programs focused on poultry husbandry, biosecurity, and responsible ABU. Moreover, cooperatives would strengthen farmers’ bargaining power by enabling bulk procurement of poultry production inputs at reduced costs, thereby enhancing affordability and promoting adherence to recommended treatment protocols.

The final challenge lies in the enforcement of existing legislation and, where necessary, developing new legal frameworks to improve ABU and its surveillance. None of the interviewees reported having witnessed any inspections related to antibiotic prescription, dispensing, or use, whether on farms, in veterinary clinics, or at agrovet shops. Several participants also noted the increasing number of unofficial agrovet outlets operating without regulation. In response, Malagasy authorities could enhance oversight of the veterinary drug distribution chain to ensure that all actors, from importers to retailers, are properly licensed, regularly inspected, and only distribute approved, quality-assured medications. Legal instruments could also be introduced to monitor antimicrobial sales and restrict over-the-counter access to antibiotics. In addition, better regulation of poultry product markets through mechanisms such as price controls and quality inspections could help stabilize farmers’ incomes and reduce their dependence on antibiotics as a tool for maximizing short-term profits [49]. At the same time, a careful balance must be struck between strengthening regulation and ensuring equitable access. For example, making antibiotics prescription-only may inadvertently limit access for underserved communities who rely on informal or distant sources for animal health care [8,27]. Regulatory reform must therefore be accompanied by support measures that expand coverage by extension services providing technical support to poultry farmers and promote responsible antibiotic use without excluding vulnerable populations.

### 4.3 Study limitations and perspectives

We sought to mitigate potential biases through the use of comprehensive data collection and analysis tools. However, our findings are derived from a convenience sample of poultry farmers, veterinary agents, and agrovet shop salespersons, which may introduce selection biases. As such, the results should be interpreted with caution and are not intended to be statistically representative of the entire Analamanga region. Instead, they provide a detailed and context-rich exploration of the perspectives and experiences of a specific subset of stakeholders within the study area.

We aimed to include participants from all three poultry farming sectors present in Analamanga, but intensive farms were relatively few in number and more difficult to access, and this lack of participation may be explained by several factors. These include a desire to preserve operational confidentiality (possibly due to concerns that the study’s findings could attract unwanted attention from veterinary or tax authorities) as well as fears that interviewers visiting multiple farms might inadvertently introduce disease. This limited engagement from major actors in the poultry sector has also been reported in similar research contexts [61,62].

Our interview guides did not include a dedicated question on ABU/ABR training for poultry farmers and animal health professionals (the latter were asked only about veterinary-drug training in general). We were also unable to provide quantitative figures for the amounts of antibiotics used and sold because our farmers interviewees did not keep written records and did not know these figures at the time of the interview. Further research should therefore be conducted with more representative samples of farmers, animal health professionals and agrovet shops, specific questions about ABU/ABR training, and a quantitative component, for example by leaving tablets on site for data collection [63]. This would allow exploring more precisely antibiotic sale and use practices and their association with potential drivers identified in our study such as the type of farm, or the source of health advice and veterinary drug procurement. While our qualitative findings suggest differences in antibiotic use practices, knowledge, and sources of advice between backyard and semi-intensive poultry farmers, these observations are exploratory and warrant further investigation through quantitative studies designed to test such associations with statistical rigor. For example, we could verify whether large semi-intensive and intensive farms are truly better positioned to invest in biosecurity, standardized protocols, and preventive measures, potentially enabling more controlled and rational ABU [49]. Or whether, their high stocking densities may increase reliance on antibiotics to manage disease risks, potentially leading to greater overall ABU [64].

## 5 Conclusion

Our findings provide the first detailed qualitative account of how poultry farmers in the highest poultry-density region of Madagascar navigate everyday production and health management challenges and make decisions regarding ABU. Our sample of backyard poultry farmers used antibiotics less systematically than semi-intensive farmers, and when they did, their practices were generally weak. They relied on advice from agrovet shop sellers rather from more knowledgeable professionals such as veterinarians and technicians, often chose the cheapest antibiotics and had very limited awareness of withdrawal periods and ABR. In our sample of semi-intensive poultry farmers, ABU practices also revealed knowledge gaps but, more notably, a widespread dependency on antibiotics driven by the precarious profitability of poultry production. This vulnerability stems from the high costs and varying quality of inputs (particularly for feed) and persistently low market prices for poultry meat and eggs. These results need to be confirmed by future research involving quantitative comparative analysis based on random sampling of farmers. They nevertheless suggest that while improving farmer awareness of preventive health measures (e.g., vaccination, biosecurity, hygiene) and antibiotic stewardship is essential, such efforts alone are unlikely to produce lasting behavioural change unless broader structural constraints including informality, weak market regulation, and limited access to financial credit are simultaneously addressed within the poultry sector.

## Supporting information

S1 File Inclusivity in global research checklist. (PDF)

S2 File Interview guides. (PDF)

S3 File Coding grids. (PDF)
