# Supplementary material for: Antibiotic stewardship challenges in Madagascar’s poultry sector: informality, knowledge gaps, and market constraints
Source: PLoS One. 2026 Nov 6;21(11):e0362299. doi: 10.1371/journal.pone.0362299 (PMC13742957; doi:10.1371/journal.pone.0362299)
Supplement: Supplementary file 1 [file pone.0362299.s001.pdf]

Inclusivity in global research

PLOS' policy on inclusivity in global research aims to improve transparency in the reporting of research performed outside of researchers' own country or community and ensures that PLOS publications reporting global research adhere to high standards for research ethics and authorship. Authors of relevant research articles may be asked to complete the questionnaire below, which outlines ethical, cultural, and scientific considerations specific to inclusivity in global research. This questionnaire may be requested when researchers have travelled to a different country to conduct research, if research uses samples collected in another country, research with Indigenous populations or their lands, or if research is on cultural artefacts. Researchers travelling to another country solely to use laboratory equipment will not normally be required to complete the questionnaire. However, the questionnaire can be requested at the journal's discretion for any submission – if you have been requested to complete this questionnaire by the PLOS journal you submitted to, please do so.

Please complete the questionnaire below and include this as a Supporting Information file with your manuscript. Note that if your paper is accepted for publication, this checklist will be published with your article in the supporting information files. Please ensure that you reference the checklist in the main body of your manuscript. We suggest adding a subsection 'Inclusivity in global research' to your Methods section and adding the following sentence: "Additional information regarding the ethical, cultural, and scientific considerations specific to inclusivity in global research is included in the Supporting Information (SX Checklist)"

The questions have been designed to be applicable to a wide range of study types, and there are subsections for both human subjects research and non-human subjects research. If any of the questions are not relevant to your research please mark them as "N/A" as appropriate.

Ethical considerations, permits and authorship

This section is applicable to all research types.

Provide details as to who granted permissions and/or consent for the study to take place in the Methods section of your manuscript. This should include the names of **all** ethics boards, governmental organizations, community leaders or other bodies that provided approval for the study. If individuals provided approval refer to these people by their role or title but do not list their name(s).

The study protocol was submitted to and approved by the Department of Veterinary Services of the Ministry of Agriculture and Livestock. It was also submitted to the Madagascar National Ethics Committee in Life Sciences and Health, which confirmed that formal ethical approval was not required for this type of study.

If there were any deviations from the study protocol after approval was obtained please provide details of these changes in the Methods section of your manuscript.

There was no deviation from the study protocol.

Did this study involve local collaborators that are residents of the country where the research was conducted or members of the community studied? If you do not have any authors from said communities, please provide an explanation for this below.

Three of the authors (Ilo Tsimok'Haja Ramahatafandry, Michel Rakotoharinome and Daouda Kassie) are residents of Madagascar.

Everyone listed as an author should meet PLOS' criteria for authorship and all individuals who meet these criteria should be included in the author byline, rather than the acknowledgements. For further information please see the journal's Authorship Policy.

Human subjects research (e.g. health research, medical research, cross-cultural psychology)

Did you obtain written informed consent from a representative of the local community or region before the research took place? How did you establish who speaks for the community? Details of written informed consent obtained from study participants should be reported separately in the Methods section of your manuscript.

We obtained written informed consent from the chef de fokontany of each fokontany where data collection took place. Fokontany represent the smallest administrative unit in Madagascar and serve as the primary governance structure at the local level. The chef de fokontany (chief of the fokontany) is the officially recognized representative of the community, responsible for overseeing administrative, social, and developmental matters within their jurisdiction. As such, they are empowered to grant permission for research activities conducted within their fokontany.

How did members of the local community provide input on the aims of the research investigation, its methodology, and its anticipated outcome(s)?

Local community members (chefs de fokontany and trusted intermediaries identified by the chefs de fokontany) actively contributed by identifying and mapping poultry farmers and agrovet shops in their community. While the study's overall aims and methodology were predefined, their input helped adapt the research to local realities, facilitated trust, and improved participation among farmers and agrovet shops workers.

When engaging with the local community, how did you ensure that the informed consent documents and other materials could be understood by local stakeholders?

All written materials were prepared in Malagasy, the national language of Madagascar. Additionally, a local guide (a trusted intermediary identified by the chef de fokontany) accompanied the research team during all interactions with farmers, agrovet shop workers and animal health professionnals. This guide played a crucial role in explaining the study's objectives, procedures, and the informed consent process in a clear and culturally appropriate manner. This approach ensured that all stakeholders, regardless of literacy level, had a comprehensive understanding of the research and their rights as participants.

Will the findings of the research be made available in an understandable format to stakeholders in the community where the study was conducted (e.g. via a presentation, summary report, copies of publications, etc.)? Please provide details of how this will be achieved.

At the conclusion of the project, a restitution meeting was held in Antananarivo to present the key findings to representatives of the veterinary services as well as other relevant stakeholders who can integrate them into their training and awareness-raising activities in the Analamanga region.
While we had initially hoped to organize restitution meetings in each of the municipalities where data collection took place, this was unfortunately not feasible due to time and budget constraints at the end of the project. We propose to prepare a summary report in Malagasy that can be shared with local veterinary services, chick and feed suppliers, and other key intermediaries who interact regularly with poultry farmers.

Non-human subjects research using specimens/ animals collected as part of the study, or those housed in archival collections. Examples include archaeology, paleontology, botany and zoology.

Did the permission you obtained from a local authority to perform the study include an agreement on access to outputs and benefit sharing? This may include procedures to enable fair distribution of the benefits and resources arising from the research performed. Please include any details of Prior Informed Consent and Benefit Sharing Agreements obtained. These may be required by field-specific regulations, for example the Convention on Biological Diversity (CBD) and the associated Nagoya Protocol.

No specimens or animals were collected as part of the study.

If the material used in your study was imported, please A) provide the year it was imported and B) indicate whether permits were obtained to import/export the materials used, C) provide details of any permits obtained. If this information is not available, please indicate this.

No material was imported as part of the study.

If you used archival specimens, please state how the material used in your study was acquired by the institute it is held in and provide details of any permits obtained for the original excavations/ sample collection. If this information is not available, please indicate this.

No archival specimens were used as part of the study.

How was the potential cultural significance of the materials collected in your study to local communities considered in your research design? Were Indigenous peoples and/or local researchers and institutions involved with archaeological excavations / collection of specimens? If so, please provide a description of their involvement.

No materials were collected as part of the study.

If your manuscript includes photographs of human remains please indicate whether authors obtained permission from descendants or affiliated cultural communities to do so.

No photographs of human remains were taken as part of our study.
