# Supplementary material for: Antibiotic stewardship challenges in Madagascar’s poultry sector: informality, knowledge gaps, and market constraints
Source: PLoS One. 2026 Nov 6;21(11):e0362299. doi: 10.1371/journal.pone.0362299 (PMC13742957; doi:10.1371/journal.pone.0362299)
Supplement: Supplementary file 2 [file pone.0362299.s002.pdf]

INTERVIEW GUIDE FOR FARMERS

Part 1: General Information

Farmer's unique ID: ………..	
Location of the farm	Antanetibe Mahazaza / Ivato / Itaosy / Ambohijanaka
What is your age?	
Gender	Male / Female
What is your level of education?	No schooling / Elementary / Secondary / Higher
What type of farming do you practice?	Traditional / Semi-intensive / Intensive
Who owns the farm?	Individual / Cooperative / Family / Other:.....
How long have you been raising livestock?	

Are you a member of a farmers' association?	Yes / No
What types of animals do you raise?	Laying hens (Number):.........<br>Broiler chickens (Number):........<br>Gasy chickens (Number):.........<br>Other poultry (Number):.......<br>Small ruminants (number):.......<br>Cattle (Number):..........
How often do you introduce new chicks?<br>How often do you introduce new pullets ready to lay?	……………………………………………<br>--
What kind of support do you receive?	None<br>Mainly private support<br>Mainly government (public) support<br>Both (private + public)

Do you regularly vaccinate your poultry?	Yes / No

Part 2: Description of the farm

1.1 To begin with, can you describe your poultry farm, including the breeds you raise and the feeding and care practices you use? Additionally, can you explain your role on the farm and how you are involved in the management and decision-making regarding your business?

- Have there been any changes recently in terms of their numbers (increase or decrease) over the past few years? Why do you think that is?
- Can you rank the reasons behind these changes in order of importance?
- Is the business going as you had planned? (Successes and challenges)
- What would you like to change or improve? (Goals for the coming years)

Follow-up questions

- Do you also have other activities besides livestock farming? Which is your main activity? What is the role of livestock farming in your overall activities in terms of income?
- How did you get started? (Where did the idea come from? What resources did you use? [source of initial funding for the activity] What do you think of the space in terms of size, whether or not you built a facility, etc.)

- How did you learn about livestock farming? Was it a tradition in your family or neighborhood, or was it new to you? Did you follow the example of others who already had livestock farms, or did you seek advice from professionals?
- What are the requirements for raising livestock that you were told about? Who told you about them?
- What documents did you need to start the farm? Who asked you for these documents?

1.2 Can you give me more information about your farm?

- What motivated your choice of the type of poultry you raise?
- How many people work with you? Who are they, and what is each person's role?
- Where and how do you sell your production? What portion is intended for personal or family consumption, for stable or informal sales channels, or for local or broader markets?)

Part 3: Farm Health and Medication Use: Assessing Care Practices on Poultry Farms

3.1. Poultry feed

- How do you feed your animals? Where do you buy their food? How much does poultry feed cost? Why do you buy it there?
- In your opinion, what foods are good for poultry and why? What is good for: weight gain, better health, etc.?
- What are the ingredients in the feed you give your animals? If you don't know, is this information you'd like to find out?

3.2. Diseases and Medications

- Where and from whom do you buy chicks/poultry? Have you stayed with the same supplier?
- When you buy chicks, do you know if they have been given medication before?
- Describe the diseases or health issues that occur most frequently on your farm. How and when do you notice them? Can you tell us the names of these diseases or describe these health issues?
- When do you use medications to treat your poultry? What are the main medications you use to treat your poultry? Do you have a preference for any of these medications?
- Do you sometimes use traditional medicines (herbs, decoctions, amulets, etc.)?

- In your opinion, what is an antibiotic?
    - Do you give them to your poultry? Can you give us some examples of names?
- In what situations are antibiotics important for poultry health? Which antibiotics do you use most frequently? Name, dose, frequency, duration of treatment, equipment used, etc.
- Do antibiotic usage protocols depend on the type of poultry being raised (chickens, turkeys, etc.)? Why?
- Who do you consult for advice regarding your farm? What justifies your choice of this person?
- Who prescribes or advises you on the antibiotics you give to the animals? Can humans take these antibiotics? Do you always purchase antibiotics with a prescription?
- Are veterinary medications available in your community? Where do you typically buy antibiotics?
- Do you have a stock of veterinary medicines and/or antibiotics? Is it possible to see it? ***For the interviewer***: Note the medicines present (brand names), quantities, take photos)

- What precautions do you take regarding the use of medications? (Keeping them out of children's reach, expiration dates, storage temperature, etc.)? Where did you obtain these instructions? Package insert, advice from veterinarians, other sources to be specified.
- How long after administering an antibiotic do you sell or consume your livestock products? Have you ever been inspected regarding this? If so, by whom? What did they tell you?
- Are there any conditions you must meet in order to sell your products? Which ones relate to antibiotics? Who sets these conditions?
- Would you prefer to use more antibiotics or fewer antibiotics? Why?
- How can the Malagasy government encourage poultry farms to use alternatives to antibiotics?

Part 4: Relationships with veterinarians or other advisors or authorities

- Do you know any veterinarians or other animal health specialists? Are they available and accessible here? What is the average cost of their services (including travel)? Is this expensive for you?
- For what procedures do you seek the help of a professional (veterinarian, livestock technician)?
- If vaccinations are involved, against which disease, for example? Did the person who administered the vaccination discuss preventive measures for certain animal diseases with you? If so, what did they tell you?
- Have you ever experienced situations where antibiotics were ineffective?
- If so, what do you think explains this lack of effectiveness?
- What do you think antibiotic resistance is? Does it only affect humans or animals? Where did you get this information?
- Has your farm ever been "inspected"? If so, why? By whom? Is it a different veterinarian from the one who comes to treat the animals?
- What are the main pieces of advice or recommendations that veterinarians, medication sellers, or authorities give you regarding your farm?
- Do you follow these recommendations? Do these recommendations seem appropriate for your situation? Why? Can you give an example of an inappropriate recommendation? And an example of a particularly appropriate recommendation?
- Who do you feel is best able to give you advice appropriate to your situation?
- Do you seek advice from other farmers?

Part 5: Environment and General Questions

- How are the poultry housed on your farm? ⇒ Type of coop, type of buildings, etc.
- How is poultry waste managed? What measures do you take for waste management (disposal, reuse, etc.)?
- How do you ensure the cleanliness of the poultry environment?
- How are the birds handled during medical procedures, cleaning, feed changes, etc.? And how are they transported within and outside your farm?
- How do you manage animal stress in general? If any products are used, please specify their names

=======================================*******================================

INTERVIEW GUIDE FOR ANIMAL HEALTH PROFESSIONALS (VETERINARIANS, TECHNICIANS)

Part 1: General Information

Professional's unique identifier: ……………..	
Location of the survey area	Antanetibe Mahazaza / Ivato / Itaosy / Ambohijanaka
What is your age?	
Gender	Male / Female
What is your level of education?	No schooling / Elementary / Secondary / Higher

What is your employment status?	Veterinarian / Veterinary technician / Other (please specify): ……………
How long have you been practicing your profession?	
Have you ever received training on veterinary drugs?	Yes / No
• If yes, by whom?	……………………………….
• If yes, for how long?	……………………………….
Do you have an assistant or a substitute who helps you care for the animals?	Yes / No
If yes, have they received training in veterinary care?	Yes / No / I don't know

Part 2: Regulations on the use of antibiotics in poultry farms

- What are the guidelines for prescribing/dispensing antibiotics to farmers?

Follow-up questions

- Do the guidelines for prescribing vary depending on the type of farm (swine, poultry, cattle, etc.)? Can you explain your answer?
- How does the government ensure that animal health professionals comply with regulations regarding the prescription and dispensing of antibiotics?
- What are the penalties for animal health professionals who do not comply with these standards?
- Have these standards changed over time? If so, what changes are you aware of?

- In your opinion, why are antibiotics important in poultry farming?

- What could be the effects on animal, human, and environmental health in the future (5 years, 10 years, or more)? In your opinion, what measures should be taken to prevent these effects?
- What measures has the government imposed on animal health professionals regarding the use of veterinary drugs in Madagascar? What is your opinion on the effectiveness of these measures? Have you ever encountered difficulties in implementing these measures? If so, what were they? How do these measures affect your daily activities?
- What penalties does the government impose if professionals fail to comply with the regulations regarding the sale of medications?
- What advice can you give to livestock farmers to reduce the use of antibiotics on their farms?
- How do you think the use of antibiotics in poultry farms could be improved to better protect animal health?
- How can you help implement the measures established by the government to promote better antibiotic use in Madagascar?
- What kind of information or advice do you provide to poultry farmers regarding the use of antibiotics in poultry? Are there other individuals, organizations, or government agencies that offer advice or training to help them better manage antibiotic use?
- In your opinion, how can poultry farmers help reduce or improve the use of antibiotics on their farms?
- Do antibiotic use protocols depend on the type of poultry being raised (chickens, turkeys, etc.)? Why?
- What is the role of animal health professionals in monitoring antibiotic use on poultry farms?
- How can you help farmers and veterinary drug sellers understand the consequences of antibiotic misuse?

=====================================*******==============================

INTERVIEW GUIDE FOR VETERINARY DRUG SELLERS (UNOFFICIAL VETERINARY DRUG OUTLETS, VETERINARY PHARMACIES)

Part 1: General Information

Unique seller ID: ……………..	
Location of the point of sale	Antanetibe Mahazaza / Ivato / Itaosy / Ambohijanaka
What is your age?	

Gender	Male / Female
What is your level of education?	No schooling / Elementary / Secondary / Higher
What is your employment status?	Veterinarian / Veterinary Technician / Salesperson / Other (please specify): ……………
How long have you been working as a veterinary drug salesperson?	
Type of retail outlet	Unofficial warehouse / veterinary pharmacy
Status of the retail outlet	Agency (company representative) / Family-owned warehouse / Warehouse for a private individual / Warehouse owned by a partner / Other (please specify): ………
Do you have a ministerial decree authorizing the opening of your retail outlet?	Yes / No
Have you ever received training on veterinary drugs?	Yes / No
• If yes, by whom?	……………………………….

• If yes, for how long?	……………………………….
Do you sell other health products in addition to veterinary medicines?	Human medications / Plant protection products / Other (specify): ………..
Do you have an assistant or a substitute who helps you with sales here?	Yes / No
If yes, have they received training on veterinary medicines?	Yes / No / I don't know

Part 2: Information on veterinary medicines

- What are your sources of supply for veterinary medicines?
- Are you satisfied with your sources of supply? (in terms of purchase cost, drug quality, selection of available drugs…)
- Do you have an idea of what percentage of your veterinary drug sales are antibiotics? Why?
- Among the medications you sell, which ones are the best sellers (please specify the name)? Why?
- How do you decide which antibiotics to sell, taking into account regulations and market demand?
- What measures must be followed before prescribing or dispensing antibiotics to livestock farmers? How do you help customers choose the most appropriate antibiotics for their animals while complying with regulations?
- What are the potential effects of antibiotic misuse on animal and human health?
- How can veterinary drug sellers in Madagascar contribute to the global effort to reduce antibiotic resistance?

Part 3: Regulations Governing the Sale of Veterinary Medicines

- What is the distribution chain for veterinary medicines?
- How do you (as veterinary drug sellers) ensure that the antibiotics you sell follow the drug distribution chain?
- What types of customers buy from you? (Technicians, livestock farmers, feed suppliers, others)

- Have there been any regulatory changes regarding the sale of veterinary medicines? If so, what are they? How have these changes affected the sale of veterinary medicines? Do you know if any of these changes pertain to antibiotics? If so, which ones?
- What procedures must be followed to sell antibiotics (prescription required, no prescription required, customer request, documentation, etc.)?
- What challenges do veterinary drug sellers face regarding the sale of antibiotics, and how do you overcome them?
- How do veterinary drug sellers collaborate with the government to ensure the quality of the antibiotics sold? What kind of collaboration is this?
- What measures has the government imposed on sellers regarding the sale of veterinary medicines in Madagascar? What is your opinion on the effectiveness of these measures?
- What penalties does the government impose if sellers fail to comply with the government's regulations regarding the sale of medications?
- What advice can you give to livestock farmers to reduce the use of antibiotics in their operations?
- Have you received training on antibiotic resistance? If so, from whom? If not, would you be interested in learning more about the topic?
