# Supplementary material for: Antibiotic stewardship challenges in Madagascar’s poultry sector: informality, knowledge gaps, and market constraints
Source: PLoS One. 2026 Nov 6;21(11):e0362299. doi: 10.1371/journal.pone.0362299 (PMC13742957; doi:10.1371/journal.pone.0362299)
Supplement: Supplementary file 3 [file pone.0362299.s003.pdf]

Supporting Information S3 Coding grids

Coding grid for interviews of farmers

Theme	Code	Description
Advice	Advice from animal health specialists	Everything related to livestock management advice received from animal health professionals: who are these professionals (for e.g., supplier technicians, veterinarians, agronomists), how available they are, how much they charge, the farmer's level of trust in them, the quality of the advice given, how frequently they are consulted (for e.g., regularly or only when there is a problem), and whether or not the farmer follows their advice
	Advice from other sources	Everything related to livestock management advice received from individuals other than animal health professionals: who they are (for e.g., other farmers, family members, friends), how available they are, how much they charge, the farmer's level of trust in them, the quality of the advice given, how frequently they are consulted (for e.g., regularly or only when there is a problem), and whether or not the farmer follows their advice
Antibiotic use	Antibiotic resistance	Everything related to whether they have heard of ABR and what their understanding of ABR is
	Definition	Everything related to what is their definition of an antibiotic
	No use	Everything related to situations when the farmers say they do not use antibiotics, including reasons why they do not use antibiotics
	Reasons	Everything related to reasons why farmers use antibiotics. Also includes responses pertaining to reasons why they wish to use more or fewer antibiotics in the future
	Source	Description of where antibiotics are purchased and whether advice is provided or a prescription is necessary when purchasing antibiotics
	Treatment failures	Everything related to possible failures observed in antibiotic treatments and the presumed reasons for these failures
	Use	Everything related to how people read and apply instructions for using antibiotics, how and by whom antibiotics are stored, administered (including the route, dosage, duration), and possibly reused (for animals or for humans)
	Withdrawal periods	Everything related to awareness or lack of awareness of withdrawal periods when using antibiotics (including any knowledge of the risks of not observing the withdrawal period) and related practices (length of the withdrawal period, sale of eggs and/or the animal, consumption)
Authorities	Authorities	All information regarding establishment permits, farm inspections, taxation, and the support the government provides/does not provide/should provide regarding livestock farming or the use of antibiotics in livestock farming
Farm	Development	Everything related to development of the farm, causes of changes in the development of the farm and current level of satisfaction with the development of the farm
	Future	Everything related to future goals and aspirations for the farm
	Importance	Everything related to importance of poultry farming compared to other occupations/sources of income

Theme	Code	Description
	Motivation	Everything related to motivations for starting poultry farming
	Problems	Everything related to problems encountered on the farm (for e.g., financial problems, lack of land available, etc.)
Human resources	Training and socialization	Any information on training on livestock farming that people working in the industry have received. Also includes “on-the-job” training by observing other farmers.
	Workers	Description of the number of people working on the farm, their status (for e.g., family member or employee), and their role
Infrastructure	Building	Everything related to the building where poultry are raised: the farmers’ home or a specific building, description of the floors, walls, roofs, etc.
	Biosecurity and Hygiene	Everything that contributes to biosecurity: equipment (for e.g., footbaths), controlled access to the farm (for e.g. entry restrictions), etc.; and everything that contributes to hygiene: regularly changing the litter, cleaning, disinfecting, ensuring proper ventilation, maintaining a downtime, etc
Inputs	Chicks	Everything related to chicks: breed, source of supply, quality (for e.g., grows well, falls sick), price, availability, and timeliness of supply
	Feed	Everything related to feed: type of feed, produced on-site or purchased ready-made, source of supply, reason for feed selection, quality (good, poor), price, availability and timeliness of supply, which feed is considered best
Poultry health care	Caregiver	Everything related to the person(s) involved in health care and the type of care provided (maintaining temperature, administering dietary supplements or medications)
	Diseases	Everything related to diseases encountered in poultry farming: name, frequency, severity, cause, symptoms, treatments, prevention, etc.
	Medications	Everything related to the products used (feed supplements and/or medications): place of purchase, any advice given by the seller, prescription or over-the-counter, storage conditions, reasons and methods of use, use of traditional remedies… Also includes information regarding when the farmer cannot recall the medications used
	Stress	Everything related to animal stress: what causes it, how it is prevented or managed
	Vaccination	Everything related to vaccination: information on the vaccination status of chicks, vaccination practices within the farm (type of vaccine, frequency of use, administration methods, etc.)
Sales	Manure	Everything related to manure sales arrangements
	Poultry and eggs	Everything related to terms of disposal of animals and eggs (personal consumption and/or sale) and characteristics (e.g., consumption of broken eggs, direct sale or through an intermediary, selling price, etc.). Also includes information on the demand for and creditworthiness of buyers of animal products

Coding grid for interviews of animal health professionals

Theme	Code	Description
Control by authorities	Effectiveness	Everything related to the effectiveness of the control of antibiotic prescription, dispensing, sale and use
	Evolution	Everything related to how the control of antibiotic prescription, dispensing, sale and use has changed over time
	None	Everything related to the perception or experience that no control is done on antibiotic prescription, dispensing, sale and use
	Penalty	Everything related to the type of sanction observed (or not) in real life when controls reveal faults in antibiotic prescription, dispensing, sale and use
	Type	Everything related to the type of control (of antibiotic prescription, dispensing, sale and use) implemented: verification of knowledge, of documentation, etc.
Factors influencing antibiotic prescription	Animal	All factors related to the animal and that influence antibiotic prescription: species, type of poultry (broiler/layer/gasy), the animal's condition and weight, etc.
	Disease	All factors related to the disease and that influence antibiotic prescription: bacterial, viral, or other disease; history of previous disease outbreaks; how the disease responded to past treatments; etc.
	Laboratory	All factors related to the veterinary testing laboratory and that influence antibiotic prescription: test results, recommendations, etc.
	Livestock farming	All factors related to the farm or the farmer and that influence antibiotic prescription: farm size, farming conditions (including biosecurity), specific requests for antibiotics, etc.
Negative effects of antibiotic use (on the health of animals, humans, and the environment)	Antibiotic resistance	Everything related to awareness of antibiotic resistance as a possible effect of antibiotic use (including the transmission of resistance)
	None	Situations when no negative effects of antibiotic use are known or have been observed
	Other	Other potentially negative effects of antibiotic use: allergy, onset of another disease, etc.
	Prevention	Everything that helps prevent the potentially negative effects of antibiotic use: adherence to antibiotic indications and dosages, proper cooking of meat, etc.
Suggestions for improvement of antibiotic use	Farmers	All suggestions and recommendations aimed at farmers to improve antibiotic use: farm hygiene, animal feeding, not treating animals without genuine professional advice, vaccination, etc.
	Healthcare Professionals	All suggestions and recommendations aimed at healthcare professionals to improve antibiotic use: follow dosage instructions, take samples in case of mortality, try to administer vitamins without antibiotics as a first-line treatment, etc.
	Others	All suggestions and recommendations aimed at individuals other than farmers and healthcare professionals (retailers, diagnostic laboratories, feed manufacturers, etc.) to improve antibiotic use:

Coding grid for interviews of agrovet shop workers

Theme	Name	Description
Antibiotic resistance and the misuse of antibiotics	Effects	Everything related to the effects of ABR and antibiotic misuse on the health of humans, animals and the environment
	Fight	Everything related to how veterinary drug sellers can contribute to the global effort to reduce ABR and misuse of antibiotics
	Training	Everything related to potential training on ABR and antibiotic misuse: whether they received training, who provided the training, interest in information on the subject
Relationship with authorities	Measures	Everything related to knowledge of measures/regulations imposed by authorities on the sale of veterinary drugs in Madagascar
	Penalties	Everything related to knowledge of penalties imposed by authorities if sellers fail to comply with regulations on the sale of veterinary drugs
	Quality	Everything related to how veterinary drug sellers can collaborate with the authorities to ensure the quality of the veterinary drugs sold
Sales of veterinary drugs	Customers	Everything related to who are the customers of the shops for veterinary drugs (technicians, livestock farmers, feed suppliers, etc.) and what they ask for (products, advice, etc.)
	Most	Which are the best-selling veterinary drugs in the shop
	AB share	Everything related to the share of antibiotics in veterinary drug sales
	AB decision	Everything related to criteria used when deciding which antibiotic to recommend and/or to sell
	AB compliance	Everything related to instructions given to customers for using antibiotics and ensuring compliance
	AB advice	Everything related to advice given to customers for reducing the use of antibiotics
	AB issues	Everything related to issues faced when selling antibiotics and strategies to overcome them
Supply channels for veterinary drugs	Knowledge	Everything related to knowledge of the distribution chain for veterinary drugs in Madagascar
	Source	Everything related to which are the shop's sources of supply of veterinary drugs
	Satisfaction	Everything related to satisfaction with the shop's sources of supply of veterinary drugs (purchase cost, drug quality, selection of available drugs, etc.)
	Verification	Everything related to potential means of verification of the quality of the source(s) of supply of veterinary drugs
